# Supplementary material for: Economic impact of a large-scale scabies upsurge on healthcare facilities in Rohingya refugee camps: A retrospective costing study
Source: PLOS Glob Public Health. 2026 Apr 3;6(4):e0005772. doi: 10.1371/journal.pgph.0005772 (PMC13048477; doi:10.1371/journal.pgph.0005772)
Supplement: Attachment — Submitted filename: PGPH-D-25-03807_R1_editor.pdf [file pgph.0005772.s003.pdf]

PLOS Global Public Health

Economic Impact of a Large-Scale Scabies Upsurge on Healthcare Facilities in Rohingya Refugee Camps: A Retrospective Costing Study

--Manuscript Draft--

**Manuscript Number:**	PGPH-D-25-03807R1
**Article Type:**	Research Article
**Full Title:**	Economic Impact of a Large-Scale Scabies Upsurge on Healthcare Facilities in Rohingya Refugee Camps: A Retrospective Costing Study
**Short Title:**	Economic Impact of a Scabies Outbreak in Rohingya Camps
**Corresponding Author:**	Charls Erik Halder, M.B.B.S., M.P.H., M.Sc. (Global health)<br>International Organization for Migration<br>Dhaka, BANGLADESH
**Order of Authors:**	Charls Erik Halder, M.B.B.S., M.P.H., M.Sc. (Global health)
	Md Abeed Hasan
	James Charles Okello
	Sayed Sunny Uz Zaman
	Julekha Tabassum Poly
	Hamim Tassdik
	Dickson Wafula Barasa
	Emmanuel Roba Soma
	Md Farhad Hussain
	U Maung Prue
	Sirajul Munir Khandokar
	John Patrick Almedia
	Jahangir Alam
**Keywords:**	Scabies; Economics; Healthcare Costs; Cost of Illness; Rohingya; Disease Outbreaks; Mass Drug Administration; Communicable Disease Control; Bangladesh
**Abstract:**	Background<br><br>Scabies is one of the common infectious skin conditions globally, with a significantly high burden in hot and tropical countries, resource-poor settings, and areas with high population density. Rohingya refugee camps in Cox's Bazar are one of the most protracted refugee crises in the world, sheltering approximately 1,143,096 refugees in 33 overcrowded refugee camps. While most existing literature focuses on mass drug administration (MDA) interventions or community-level estimates, the economic burden of scabies on health system is rarely studied and documented.<br><br>Methods<br><br>This is a retrospective cost study, where we have used financial and epidemiological data from January 2021 to December 2024. Costing was done from the provider's perspective, focused on what International Organization for Migration (IOM) spent during the period as a health service provider. A combination of standard stepdown approach and micro-costing methods were used. Financial data for the study period 2021 to 2024 were collected from the IOM health programme's annual budget and consumption reports. The study population included all individuals who were clinically diagnosed with scabies and received care at 35 IOM-supported health facilities in Ukhiya and Teknaf, Bangladesh.<br><br>Results

Powered by Editorial Manager® and ProduXion Manager® from Aries Systems Corporation

	The overall estimated financial cost for IOM's scabies outbreak response through its health facilities was USD 2.12 million, with an annual average of USD 531,729. The average cost per scabies management ranged between USD 5.33 and USD 6.54, with the highest in 2021 and the lowest in 2022. Drug costs accounted for 11.92% of the overall cost over 4 years. Of the total cost of $253,629.43 over 4 years, 79% was attributed to permethrin topical cream, which was used to manage an estimated 85% of the total managed cases. Scenario analysis demonstrates that the existing permethrin-based treatment preference is the most expensive treatment modality, compared with ivermectin-based treatment and mixed-treatment approaches.<br><br>Conclusion<br><br>Although the average cost of treating scabies is relatively low, overall, the treatment cost for such a large population has a significant economic impact. This study found a substantial effect of mass drug administration on reducing the financial burden on the healthcare system.
**Opposed Reviewers:**	
**Additional Information:**	
**Question**	**Response**
**Financial Disclosure**<br><br>Enter a financial disclosure statement that describes the sources of funding for the work included in this submission and the role the funder(s) played. This includes grants and any commercial funding of the work or authors.<br><br>This statement will be typeset if the manuscript is accepted for publication.<br><br>*Please review the submission guidelines and the instructions link below for detailed requirements and guidance.*	The authors received no specific funding for this work. The study was conducted as part of routine public health programme activities of the International Organization for Migration (IOM) in Cox's Bazar, Bangladesh. All authors were salaried employees of IOM at the time of the study. No external grants, commercial funding, or institutional research funds were received for this work. The funders had no role in study design, data analysis, decision to publish, or preparation of the manuscript.
**Competing Interests**<br><br>On behalf of all authors, disclose any competing interests that could be perceived to bias this work.<br><br>This statement will be typeset if the manuscript is accepted for publication.<br><br>*Please review the instructions link below*	The authors declare no competing interests. This study received no external funding. The authors used generative AI tools only for language editing and improving clarity during manuscript preparation. All scientific content, data analysis, and conclusions were developed solely by the authors.

Powered by Editorial Manager® and ProduXion Manager® from Aries Systems Corporation

and PLOS Global Public Health's competing interests policy to determine what information must be disclosed at submission.	
**Data Availability**<br><br>Before publication, Authors are required to make fully available and without restriction all data underlying their findings. Please see our PLOS Data Policy page for detailed information on this policy.<br><br>A **Data Availability Statement**, detailing where the data can be accessed, is required at first submission. Insert your Data Availability Statement in the box below.<br><br>Please see the data reporting section of our submission guidelines for instructions on what you need to include in your Data Availability Statement.<br><br>This statement will be typeset if the manuscript is accepted for publication.<br><br>*PLOS allows rare exemptions to address legal and ethical concerns. If you have legal or ethical restrictions, please use the box below to detail these in full sentences for the Journal team to consider.*	All data underlying the findings of this study and the analysis files are publicly available on Zenodo (DOI: https://doi.org/10.5281/zenodo.17697474). The repository contains fully anonymized, aggregated scabies case counts and the costing inputs used in the economic analysis for 2021–2024. No individual-level or potentially identifiable participant data are shared. Related datasets from the same scabies research series are available on Zenodo (DOIs: https://doi.org/10.5281/zenodo.17648621 and https://doi.org/10.5281/zenodo.17687717).

Powered by Editorial Manager® and ProduXion Manager® from Aries Systems Corporation

Dear Editors,
PLOS Global Public Health

We are pleased to submit our manuscript entitled **"Economic Impact of a Large-Scale Scabies Upsurge on Healthcare Facilities in Rohingya Refugee Camps: A Retrospective Costing Study"** for consideration in *PLOS Global Public Health*. This manuscript presents the **first cost analysis of a massive scabies outbreak in a humanitarian emergency setting**, using four years of financial and epidemiologic data from 35 IOM-supported health facilities in Cox's Bazar, Bangladesh.

Scabies outbreaks are common in overcrowded, resource-limited settings, yet **their economic burden on health systems remains largely undocumented**, creating a critical evidence gap for policymakers. Our findings highlight that scabies management, while inexpensive per case, results in a substantial cumulative financial burden in large-scale outbreaks—totaling **USD 2.12 million between 2021 and 2024**. We also demonstrate the **cost-saving effects of mass drug administration (MDA)** and identify a more cost-efficient treatment regimen through scenario analysis.

This research directly supports PLOS Global Public Health's mission by addressing **health systems strengthening, infectious disease control, humanitarian health delivery, and cost-effectiveness in low-resource settings**. The data can guide national and international agencies planning scabies interventions, MDA campaigns, and resource allocation in refugee and densely populated communities.

We confirm that:

1. This manuscript has not been published and is not under consideration elsewhere.
2. All authors have approved the final version and consent to submission.
3. Ethical approval was obtained from the Cox's Bazar Medical College Ethical Review Board (Ref: CoxMC/2023/017).
4. The study used anonymized, aggregated retrospective data with no individual identifiers.
5. Authors declare no competing interests.
6. Data and code have been deposited in Zenodo and are openly accessible.

We believe this manuscript will be of strong interest to researchers, global health practitioners, and policymakers working in **neglected tropical diseases, humanitarian health, outbreak control, and economic evaluation**.

Thank you for considering our submission. We appreciate the opportunity to contribute to the global evidence base on scabies control and health system resilience.

Sincerely,
Charls Erik Halder (Corresponding Author)
Migration Health Division
International Organization for Migration (IOM), Bangladesh
Email: cehalder@iom.int

Manuscript Click here to access/download;Manuscript;Manuscript.docx

Economic Impact of a Large-Scale Scabies Upsurge on Healthcare Facilities in Rohingya Refugee Camps: A Retrospective Costing Study

Charls Erik Halder[1]*, Md Abeed Hasan[1], James Charles Okello[1], Sayed Sunny Uz Zaman[1], Julekha Tabassum Poly[1], Hamim Tassdik[1], Dickson Wafula Barasa[1], Emmanuel Roba Soma[1], Md Farhad Hussain[3], U Maung Prue[1], Sirajul Munir Khandokar[1], John Patrick Almedia[2], Jahangir Alam[1]

[1]Migration Health Division, International Organization for Migration (IOM), Bangladesh
[2]Global Migration Health Research & Epidemiology Unit, International Organization for Migration (IOM)
[3]Cox's Bazar Medical College Hospital, Cox's Bazar, Bangladesh

* Corresponding author
Email: cehalder@iom.int

ABSTRACT

Background: Scabies is one of the common infectious skin conditions globally, with a significantly high burden in hot and tropical countries, resource-poor settings, and areas with high population density. Rohingya refugee camps in Cox's Bazar are one of the most protracted refugee crises in the world, sheltering approximately 1,143,096 refugees in 33 overcrowded refugee camps. While most existing literature focuses on mass drug administration (MDA) interventions or community-level estimates, the economic burden of scabies on health system is rarely studied and documented.

Methods: This is a retrospective costing study, where we have used financial and epidemiological data from January 2021 to December 2024. Costing was done from the provider's perspective, focused on what International Organization for Migration (IOM) spent during the period as a health service provider. A combination of standard stepdown approach and micro-costing methods were used. Financial data for the

study period 2021 to 2024 were collected from the IOM health programme's annual budget and consumption reports. The study population included all individuals who were clinically diagnosed with scabies and received care at 35 IOM-supported health facilities in Ukhiya and Teknaf, Bangladesh.

Results: The overall estimated financial cost for IOM's scabies outbreak response through its health facilities was USD 2.12 million, with an annual average of USD 531,729. The average cost per scabies management ranged between USD 5.33 and USD 6.54, with the highest in 2021 and the lowest in 2022. Drug costs accounted for 11.92% of the overall cost over 4 years. Of the total cost of $253,629.43 over 4 years, 79% was attributed to permethrin topical cream, which was used to manage an estimated 85% of the total managed cases. Scenario analyses demonstrates that the existing permethrin-based treatment preference is the most expensive treatment modality, compared with ivermectin-based treatment and mixed-treatment approaches.

Conclusion: Although the average cost of treating scabies is relatively low, overall, the treatment cost for such a large population has a significant economic impact. This study found a substantial effect of mass drug administration on reducing the financial burden on the healthcare system.

KEY WORDS

Scabies; Economics; Healthcare Costs; Cost of Illness; Rohingya; Disease Outbreaks; Mass Drug Administration; Communicable Disease Control; Bangladesh

INTRODUCTION

Scabies is one of the common skin conditions globally with significantly high burden in hot and tropical countries, resource poor settings and in areas with high population density. According to World Health Organization, the global incidence of scabies at any given time is more than 200 million, affecting 5 to 50

per cent of children in resource-poor areas (1). Refugees and the displaced population possess a higher risk of scabies outbreak due to overcrowded living arrangements, insufficient access to water, sanitation, and laundering facility, limited access to healthcare services and shortage of essential medicines (e.g. scabicides) (2,3).

Scabies is a curable condition, effectively treatable with the topical application of permethrin or oral administration of ivermectin. Unlike non-communicable diseases or high burden communicable diseases, such as, Tuberculosis, HIV and Hepatitis, uncomplicated scabies does not require long-term treatment. The treatment cost of scabies, if considered as single unit, is relatively inexpensive. However, scabies has the high potentiality to cause massive outbreak, especially in overcrowded and refugee settings. Overlooking its collective economic burden in an outbreak setting may create misperception among ~~the~~ policy makers and public health professionals regarding its potential economic impact and the need for adequate planning of resources. For instance, a study in Fiji reported that although the average cost per presentation for scabies at primary healthcare was US$17.7, the estimated annual healthcare costs of scabies and its complications was US$3.0 million equivalent to per capita cost of US$3.3 (4).

The Rohingya refugee camps in Cox's Bazar is one of the largest protracted refugee crises in the world sheltering approximately, 1,143,096 refugees in 33 overcrowded refugee camps (5). Overcrowded living arrangements, fragile shelters, inadequate water, sanitation, and hygiene (WASH) facilities together with heavy monsoons in both the refugee camps and neighboring host communities often result in outbreak of infectious diseases, diphtheria, measles, COVID-19, and Acute Watery Diarrhea (AWD)/cholera. In the first study of our research series on scabies in this setting, we reported a massive outbreak that occurred between 2021 and 2024, with 384,852 cases and an overall attack rate of 5,562.59 cases per 10,000 population over the four-year period (Halder et al.). This high burden increased demand for clinical services and additional resources. Simultaneously, it contributed to disruptions in daily life, potentially hindering social interactions and economic activities within the refugee community. In response to the surge of scabies cases, a World Health Organization (WHO)-supported mass drug administration (MDA) campaign was implemented in the Rohingya refugee camps between November 2023 and January 2024. The campaign targeted

approximately one million refugees and used oral ivermectin as the primary intervention. The MDA was coordinated with the Government of Bangladesh and implemented through health sector partners operating in the camps, including humanitarian agencies. This large-scale intervention aimed to rapidly reduce transmission and prevent further escalation of the outbreak (6). The disease burden of scabies infestation, along with its associated complications, places a significant financial burden on healthcare systems. It not only impacts the costs associated with medicines (e.g., topical scabicides, antibiotics for secondary infections) and diagnostics but a huge strain over spacing, infection prevention and control, health workforce and quality of service exhausting the available resources of an already overstrained health system (7).

Scabies also can lead to secondary bacterial infection, which could be complicated to life-threatening conditions, like acute glomerulonephritis and rheumatic heart fever, further efforts of healthcare workers and cost of the health system for managing the complications (8). While most existing literature focuses on mass drug administration (MDA) interventions or community-level estimates, economic burden of scabies on health facility is rarely studied and documented (4,9). However, understanding the direct healthcare costs of scabies is critical for effective planning of intervention, resource allocation and initializing cost-effective solutions, like MDA. To address this research gap, this study evaluated the financial impact of the massive upsurge of scabies on healthcare system. This research will assist the health programme and policy makers to understand financial implication of scabies upsurge and thus, facilitate proper planning for effective integration of scabies prevention and management within the essential health service model. This will also assist to examine potential impact and cost effectiveness of new prevention and management strategies or programme.

METHODOLOGY:

ETHICS STATEMENT

This study was conducted under a pre-approved protocol for secondary analysis of outbreak-related health and programme data. Ethical approval for the study protocol was obtained from the Ethical Review Board

of Cox's Bazar Medical College Hospital (CoxMC/2023/017). Administrative permission to use outbreak-response data was obtained from the Office of the Civil Surgeon and the Refugee Relief and Repatriation Commissioner (RRRC), and internal approval was obtained from the International Organization for Migration (IOM) Migration Health Research Unit.

The data analyzed in this study were routinely collected as part of public health outbreak response and health service delivery activities between 2021 and 2024, and were subsequently used for research purposes under the approved secondary-use protocol. Epidemiological case data were extracted from routine outbreak surveillance databases, while financial data were obtained from programme budget and expenditure records. The research team accessed only anonymized, aggregated numerical data and did not have access to identifiable individual-level information at any stage of the study. Given the retrospective nature of the study and the exclusive use of anonymized, aggregated data, individual informed consent was not required.

STUDY SETTING AND POPULATION

The study was implemented in Rohingya refugee camps in Ukhiya and Teknaf in Cox's Bazar. The study population included all individuals who were clinically diagnosed with scabies and received care at 35 IOM-supported health facilities in Ukhiya and Teknaf, Bangladesh, between 1st January 2021 and 31st December 2024. Scabies caused a massive upsurge during this period in the refugee camps that prompted a substantial response from IOM. Therefore, these camps were selected for this study to get an in-depth information of the outbreak.

All scabies cases were diagnosed clinically at 35 IOM supported facilities in Ukhiya and Teknaf. MSF clinical guidelines – diagnosis and treatment manual was used for diagnosis and clinical management of the cases. 5% permethrin cream was mainly used for treatment of scabies.

STUDY DESIGN

This is a retrospective costing study, where we have used financial and epidemiological data from January 2021 to December 2024. For this retrospective analysis, financial data and de-identified, aggregated epidemiological data were accessed for research purposes between 05/01/2025 and 20/02/2025. Costing was done from provider perspective focused on what IOM spent during the period as health service provider. A combination of standard stepdown accounting and micro-costing methods were used (10,11).

Micro-costing (or ingredient-based costing) was calculated direct cost per-case by itemizing resources used per patient (12). In this study, we estimated the cost per patient for medicines required, laboratory cost, and supplies for infection prevention and control.

Step-down costing method was used to estimate costing related to capitals, coordination, supervision, operation and maintenance, capacity building, risk communication and community engagement, volunteer payment and salaries and benefits of healthcare workers, meaning that total costs and resources utilized by the whole health programme of IOM during the period was systematically and proportionately distributed to the "specific service unit". The specific service unit refers, hereby, the scabies consultation and treatment service (13).

The study did not take account of the economic costs (value of resources that could have been productively used elsewhere) such as the value of volunteer time, donated goods, shared infrastructure in the scabies management. All staff and volunteers involved were salaried under the program, and all supplies, equipment, and logistics were directly financed by IOM.

DATA COLLECTION AND MANAGEMENT

Financial data for the study period 2021 to 2024 were collected from the IOM health programme's annual budget and consumption reports. A cost recording and data analysis tool was developed to record and analyze all relevant costs associated with scabies response (S1 File). Field visits to health facilities and meetings with healthcare workers and medical logisticians were made to develop the cost recording and data analysis tool. All costs were categorized by a) capital investment and b) recurrent costs. Financial and

epidemiological data were accessed for research purposes between 05/01/2025 and 20/02/2025 as part of this retrospective analysis. The research team accessed only de-identified, aggregated data and did not have access to any personally identifiable information during or after data extraction and analysis.

For shared resources, for instance, human resources (healthcare workers and volunteers), coordination, operations and maintenance, and laboratory costs, annual costs were estimated based on the total expenditure reported in the financial documents and relevant procurement orders of the programme. The total costs were then proportionally allocated for scabies using the morbidity burden of scabies in comparison to the total outpatient consultations (Table 1). Scabies response specific investment includes capacity building of healthcare workers and community health workers and development and printing of information, education and communication materials.

Table 1. Year-wise case load and proportional morbidity of scabies

	2021	2022	2023	2024
Scabies	32,046	140,590	150,470	61,746
Infected scabies	2,083	7,348	10,577	2,312
Proportional morbidity (over total consultation)	0.04	0.11	0.12	0.05
**Total outpatient consultations (all causes)**	893,925	1,302,029	1,275,773	1,126,522

Note: Proportional morbidity was calculated as scabies consultations divided by total outpatient consultations (all causes) for each year.

Capital costs included biomedical and clinical equipment used for scabies patient management, and costs related to health facility construction/renovation. The costs were annualized using the formula below, assuming the lifespan of construction for 20 years and clinical equipment for 3 years (Creese and Parker, 1994; MSF).

Annual Depreciation Cost = Useful Life (in years)/Purchase Cost of Asset

Coordination costs include salaries of programme staff and office costs at Cox's Bazar and IOM standard 7% overhead cost over the total operation and staff cost. Supervision cost includes salaries of supervisory staff, e.g. clinical supervisors, health facility in-charges and national officers. The cost of drugs was calculated on per case basis by considering the number of drugs given per patient according to standard clinical protocol and procurement prices as specified in the procurement order.

Data management and cost calculations were performed using Microsoft Excel (Microsoft Corporation, USA). The following assumptions were used to estimate drug and laboratory costs (Table 2). The percentage of case load utilizing a specific medicine was determined by reviewing the consumption report. A 3% discounting rate was used to factor for inflation across year, aligning with WHO guidance (14).

Table 2. Basis and assumptions of calculating costing of drugs and laboratory tests

Category	Type of medicine	% of case load (based on consumption report)	# of items per case	Unit price (BDT)
**Drugs**	Permethrin 5% Ointment	85.00%	2	35
	Ivermectin tab	10.00%	2	10
	Flucloxacillin 500mg cap	5.00%	20	4.8
	Flucloxacillin 250mg cap	2.00%	20	3.1
	Flucloxacillin syp 125mg/5ml	3.00%	1	50
	Cetrizine tab	64.00%	5	0.82
	Cetrizine syp	36.00%	1	9.2
	Benzyl Benzoate 25% ointment	5.00%	0.5	36

**Laboratory tests**	Renal function test, 2% of Infected case, 150 test per unit	0.12%	0.06	2,960
**IPC supplies**	Gloves	100%	2	
	Mask	5%	1	
	Hand Sanitizer (1 bottle per 1000 case)	100%	0.001	

Note: Unit prices are in BDT (Bangladeshi Taka). Permethrin 5% cream is commonly supplied as a 30 g tube; standard instruction was two applications, one week apart, and two tubes were issued per patient visit to support household contact treatment. Flucloxacillin syrup (125 mg/5 mL) is commonly supplied as a 100 mL bottle. Cetirizine tablets are 10 mg per tablet; cetirizine syrup (5 mg/5 mL) is commonly supplied as a 60 mL bottle. IPC unit prices were calculated per item based on procurement unit costs; for reference, common retail unit prices in Bangladesh include gloves (BDT 25 per pair), surgical mask (BDT 5 per piece), and hand rub/sanitizer 500 mL (about BDT 205 per bottle)

Operation and maintenance costs for health facilities included repair and maintenance, rental of land and spaces, generator and utilities, fuel, vehicle maintenance and security cost. Risk Communication and Community Engagement (RCCE) include costing for organizing awareness raising events, focus group discussions, courtyard sessions, and information, education and communication materials dedicated for scabies prevention and management. Salaries and benefits are counted for healthcare workers engaged in providing outpatient services, including triage, consultation, nursing and medication/pharmacy. Remuneration was costed for community health workers (CHW) engaged in RCCE and facility-based volunteers engaged in triage, counselling and crowd control.

Scenario analyses

A scenario-based costing analysis was conducted to measure the impact of different choice of treatment on the costing of scabies management at health facilities to find out the best approach for cost-efficient treatment of scabies. Scenarios were developed by the public health experts of IOM and based on different proportions of supply availability of permethrin, ivermectin and benzyl benzoate (Table 3). All other capital and recurrent costs were held constant to isolate the effect of different level of supply availability on per-case patient costing.

Table 3. Different planning scenario of cost analyses based on different proportion of supply availability of scabicidal agents

	Scenario I (Current)	Scenario II	Scenario III
	Permethrin-based	Ivermectin-based	Equal permethrin & Ivermectin
**Permethrin**	85%	15%*	47.5%
**Ivermectin**	10%**	80%	47.5%
**Benzyl Benzoate*** **	5%	5%	5%

* Required to manage children <15 kg and pregnant women

** Required to manage complicated cases

*** Kept constant throughout as back up to support in case rupture of both permethrin and ivermectin

In current practice (scenario 1), each patient receives two tubes of permethrin at their first visit in the clinic and based on discussion with clinicians, roughly two family members per household visit the clinics. Thus, a family of four members of different age groups roughly receives four permethrin tubes, which are shared across the members for two applications in one week apart. Ivermectin tablets are reserved only for patients with secondary bacterial infections.

In scenarios 2 and 3, since Ivermectin covers regular scabies cases (not only infected), to bring household contacts under treatment one patient is allocated on average 5 Ivermectin tablets of 6 mg with the same assumption that roughly two members per family accesses the health service at health facility.

RESULT

T[illegible]le 4: Estimated retrospective costing analysis for scabies outbreak response (2021 – 2024)

COST CATEGORIES	Type of cost	Costing approach	2021		2022		2023		2024		4 YEARS TOTAL	
			USD	% of total	USD	% of total	USD	% of total	USD	% of total	USD	% of total
**A. CAPITAL COSTS**	Biomedical & Clinical equipment	Step down	$4,587.71	2.19 %	$20,126.88	2.68 %	$21,541.30	2.65 %	$8,839.56	2.49 %	$55,095.46	2.59 %
	Health facility construction/renovation	Step down	$16,687.54	7.96 %	$50,263.58	6.71 %	$54,903.02	6.75 %	$25,514.60	7.19 %	$147,368.75	6.93 %
**SUBTOTAL**			**$21,275.25**	**10.15 %**	**$70,390.47**	**9.39 %**	**$76,444.32**	**9.40 %**	**$34,354.17**	**9.68 %**	**$202,464.21**	**9.52 %**
**B. RECURRENT COSTS**	Supervision	Step down	15,755.52	7.52 %	47,456.30	6.33 %	51,836.61	6.38 %	24,089.58	6.79 %	$139,138.01	6.54 %

	Healthcare workers remuneration	Micro-costing	$35,226.57	16.81%	$154,543.56	20.62%	$165,404.15	20.35%	$67,874.29	19.13%	$423,048.56	19.89%
	Volunteer remuneration (CHWs and facility-based volunteers)	Step down	$21,079.00	10.06%	$63,490.84	8.47%	$69,351.18	8.53%	$32,228.97	9.08%	$186,150.00	8.75%
	Drugs	Micro-costing	$21,119.31	10.08%	$92,653.18	12.36%	$99,164.41	12.20%	$40,692.53	11.47%	$253,629.43	11.92%
	IPC supplies	Micro-costing	$8,958.46	4.27%	$39,301.93	5.24%	$42,063.89	5.17%	$17,261.09	4.86%	$107,585.38	5.06%

	Laboratory consumables	Micro-costing	$1,922.76	0.92%	$8,435.40	1.13%	$9,028.20	1.11%	$3,704.76	1.04%	$23,091.12	1.09%
	Health facility – operation and maintenance (including repair, rental, utilities, vehicle, fuel)	Step down	$30,973.23	14.78%	$93,292.67	12.45%	$101,903.77	12.54%	$47,356.86	13.35%	$273,526.53	12.86%
	Capacity building	Step down	$2,000.00	0.95%	$4,120.00	0.55%	$4,243.60	0.52%	$4,370.91	1.23%	$14,734.51	0.69%
	Risk Communication and Community Engagement	Step down	$2,916.26	1.39%	$2,938.94	0.39%	$5,832.52	0.72%	$1,035.11	0.29%	$12,722.83	0.60%

	(events, sessions, IEC materials)											
**SUBTOTAL**			**$139,951.11**	**66.77%**	**$506,232.82**	**67.53%**	**$548,828.32**	**67.52%**	**$238,614.11**	**67.24%**	**$1,433,626.36**	**67.40%**
SERVICE DELIVERY AND OPERATIONAL EXPENSES (Capital Costs + Recurrent Costs)			$161,226.36	76.92%	$576,623.29	76.92%	$625,272.64	76.92%	$272,968.28	76.92%	$1,636,090.56	76.92%
**C. COORDINATION COST**	Programme staff, office costs and overhead costs (23.08% of service delivery and operational expenses)	Step down	**$48,367.91**	**23.08%**	**$172,986.99**	**23.08%**	**$187,581.79**	**23.08%**	**$81,890.48**	23.08%	$490,827.17	23.08%
**TOTAL ANNUAL COST**			**$209,594.27**	**100.00%**	**$749,610.27**	**100.00%**	**$812,854.43**	**100.00%**	**$354,858.76**	**100.00%**	**$2,126,917.73**	**100.00%**
**COST PER CASE**			**$6.54**		**$5.33**		**$5.40**		**$5.75**		**$5.53**	

227

Table 4 demonstrates the annual cost breakdown of scabies outbreak response from the years 2021 to 2024 at IOM-supported health facilities in the Rohingya refugee camps and host communities in Cox's Bazar. The overall estimated financial cost for IOM's scabies outbreak response through its health facilities was USD 2.12 million with an annual average of USD 531,729.

Aligning with the rise of scabies cases from 2021 to 2023, annual cost of scabies-related health service delivery increased four-fold from USD 209,594 in 2021 to USD 812,854 in 2023. Following the MDA campaign, the cost sharply decreased to $354,858.76 in 2024. The outbreak peak period (2022–2023) had an average annual cost of USD 781,232.35, compared with USD 209,594.27 in the pre-surge year (2021) and USD 354,858.76 in the post-MDA year (2024). The average cost per scabies management ranged between USD 5.33 to USD 6.54, with the highest in 2021 and lowest in 2022.

Healthcare worker remuneration made up 19.89% of the overall cost, consistently sharing the largest portion of the annual cost across the years, ranging from USD $[illegible]35,226 (in 2021) to $165,404.15 (in 2023). In addition to this cost, $186,150 was required for the remuneration of community health workers and health-facility-based volunteers, which is another 8 – 10% of the annual cost.

The drug costs consisted 11.92% of the overall cost in 4 years. It increased from USD 19,906.98 (11.37%) in 2021 to a peak of USD 99,164.41 (14.08%) in 2023, before dropping to USD 41,913.31 (14.12%) in 2024. The cost recording and data analysis tool used for the retrospective costing calculations is provided in the Supporting Information (see S1 File). Of the total cost of $253,629.43 in 4 years, 79% of cost was for permethrin topical cream being used for managing an estimated 85% of the total managed cases. On the other hand, tablet ivermectin only accounted for 2.58% of the drug cost, used for management of an estimated 10% of the caseload.

Additionally, an estimated USD 107,585 was utilized for infection prevention and control and USD 23,091 was utilized for laboratory consumables, making up 5% and 1% of the overall cost in 4 years, respectively. Operational and maintenance expenses for health facilities accounted for an estimated 12 to 15% of the annual cost. This proportion was calculated based on the contribution of scabies to the total number of consultations managed by these facilities.

Investment in risk communication and community engagement and capacity building were relatively low throughout the period, each category sharing less than 1% of the overall annual budget.

Table 5. SCENARIO ANALYSIS

	SCENARIO-1	Ratio	SCENARIO-2	Ratio	SCENARIO-3	Ratio
**DRUG COST**	$253,629.43	1	$211,983.90	83.58%	$ 238,718.36	94.12%
**NON-DRUG COST**	$1,873,288.30	1	$1,860,794.64	99.33%	$1,868,814.98	99.76%
**TOTAL COST**	$2,126,917.73	1	$2,072,778.54	97.45%	$2,107,533.34	99.09%

A comparative analysis of different scenarios of proportions of patients treated with topical permethrin and oral Ivermectin.

It demonstrates that existing permethrin-based treatment preference is most expensive treatment modality (Table 5 and Figure 1). Using ivermectin as the preferred treatment (scenario 2) drug costs can be reduced to nearly 83.5% of a permethrin-based approach, saving 2.55% of the overall budget. In case both drugs are utilized in equal proportion (scenario 3), drug costs drop to 94.12% of the current practice with an overall 1% total cost reduction.

Figure 1. Scenario-wise cost comparison of scabies response – Comparison of total, drug, and non-drug costs across three costing scenarios. Drug costs represent expenditures on scabicidal medicines, while non-drug costs include human resources, operations, logistics, coordination, and other programme-related expenditures. All costs are presented in United States dollars (USD).

DISCUSSION

This is the first-ever study in the humanitarian and low-resource setting which explored the economic impact of scabies in health system. The study revealed a high economic impact of scabies on the health

system valuing USD 2.12 million over four years with an average treatment cost of USD 5.53 per case. The study also found significant impact of mass drug administration on reducing economic burden on health system.

The average cost per scabies management ranged between USD 5.33 to USD 6.54. Since there was no other cost-analysis study available for scabies at humanitarian crises and massive outbreak setting, we could not compare our results with similar settings. However, the cost in our refugee setting was lower than the hospital setting in India and the primary care setting in Fiji, where the estimated average cost per patient were USD 12.20 and USD 17.7, respectively. The cost per patient also seems lower than other health burden, like tuberculosis, HIV, Hepatitis and non-communicable diseases (4,15). However, if we consider the massive scale of the outbreak, the overall financial cost of the scabies upsurge had significant impact on the health system in the low resource setting.

We have found that the estimated financial cost for the IOM's scabies outbreak response was USD 2.12 million from 2021 to 2024 with an annual average of USD 531,729. This annual average is 3.33% of the overall funding appeal made by IOM for in health programme in 2024, indicating a significant economic impact of scabies in health response (16). While IOM covers only one-fourth of the health facilities in the refugee camps, extrapolating it to sector wide response may cost around USD 2.1 million for full coverage of the catchment population in a year, which is around 2.5% of the appeal made by the health sector under the Joint Response Plan in 2025 (17). Our finding coincides with the findings of the country-level recent study of Fiji, where the researchers found although a cost measure of single case of scabies is low, extrapolating the finding across the country, the estimated cost of scabies on the Fijian health system was ~USD 3.0 million per year, equivalent to USD 3.3 per capita (4).

Our study found that annual cost for scabies outbreak response was drastically reduced in 2024 followed by the Mass Drug Vaccination campaign (November 2023 – January 2024), which was 43% of the previous year. Our previous study found that MDA contributed to a significant reduction of scabies cases, which sustained for six months followed by an upward shifting *(Halder et al)*. Aligning with this, our cost-analysis also suggests MDA contributes to significant reduction of economic burden in health systems in high

prevalence context. While the estimated cost saving following MDA in IOM facilities was USD 457,996, stimulating it to sector-wide response may lead to a saving of approximately USD 2.0 million with an investment of USD 1M required for the campaign (6). The effectiveness and cost-efficiency of Mass Drug Administration have also been noted in other settings, like Fiji and Ethiopia (9,18). In overcrowded and high burden settings, where the risk factors cannot be addressed properly, by treating the entire community at a time, MDA allows to substantially reduce the scabies prevalence and associated costs. The effectiveness of MDA will only be applicable until the scabies prevalence remains >10% of the community members (18).

We have studied three scenarios to explore the most cost-effective treatment regimen for scabies with first scenario demonstrating the existing practice of permethrin-based treatment, second scenario with ivermectin-based treatment and a third scenario where equal proportion of cases are managed by ivermectin and permethrin. Our analysis found that having ivermectin as preferred treatment (scenario 2) can reduce 16.5% of the drug cost and 2.55% over the overall scabies-related direct and indirect cost. a permethrin-based approach. When both drugs are utilized in equal proportion, 5.88% of drug costs can be reduced, while there will only be a reduction of 1% of the total cost. Topical permethrin and oral ivermectin are both effective and recommended treatment regimen for scabies, although ivermectin has contraindications in case of pregnancy and infants (1,13). A few studies suggest that topical permethrin is clinically more effective than oral ivermectin (7). In India, it was found that although the total cost of treatment with oral ivermectin was lower than topical permethrin, cost for relieving itching and transportation was higher than permethrin (19). In endemic and high-burden setting, ivermectin can be more effective if there remains significant concern on the compliance (18). Therefore, the decision of preferred treatment should not only be decided based on this economic analysis presented here, but also consideration of endemicity, compliance, clinical outcome and response time.

This was a retrospective costing analysis, therefore, in case unavailability of exact data, the researchers had to estimate the nearest possible cost based on the discussion on the relevant stakeholders. Another limitation was our study did not measure the economic impact of scabies from the patient and social perspective. For

instance, this massive scabies outbreak may have impact on the quality of life and result in social exclusion and stigma and absenteeism at school and workplace (20). There could have costs of scabies related to productivity loss, time of caregiving and travel. Therefore, while further research is warranted to understand the overall impact of scabies on health system and community, prevention and control strategies, including mass drug administration, should be imposed to reduce the burden of scabies on health system and people's lives (1).

CONCLUSION

Scabies has a high impact on health-related quality of life. Our results indicated that, although the average cost of treating scabies is relatively low, overall, the treatment cost for such a large population has a significant economic impact. This study found a substantial effect of mass drug administration on reducing the financial burden on the healthcare system. Strengthening mass drug administration and reinforcing preventive measures is crucial to prevent such an upsurge, especially in low-resourced settings like that of the Rohingya refugee camp.

FUNDING

The authors received no specific funding for this work. The study was conducted as part of routine public health programme activities of the International Organization for Migration (IOM) in Cox's Bazar, Bangladesh. All authors were salaried employees of IOM at the time of the study. No external grants, commercial funding, or institutional research funds were received for this work. The funders had no role in study design, data analysis, decision to publish, or preparation of the manuscript.

COMPETING INTEREST

The authors declare no competing interests. This study received no external funding. The authors used generative AI tools only for language editing and improving clarity during manuscript preparation. All scientific content, data analysis, and conclusions were developed solely by the authors.

DATA AVAILABILITY

All data underlying the findings of this study and the analysis files are publicly available on Zenodo (DOI: https://doi.org/10.5281/zenodo.17697474). The repository contains fully anonymized, aggregated scabies case counts and the costing inputs used in the economic analysis for 2021–2024. No individual-level or potentially identifiable participant data are shared. Related datasets from the same scabies research series are available on Zenodo (DOIs: https://doi.org/10.5281/zenodo.17648621 and https://doi.org/10.5281/zenodo.17687717).

ACKNOWLEDGMENTS

CEH conceptualized and designed the study, provided overall methodological guidance, and led the drafting and critical revision of the manuscript. MAH assisted in refining the study design and drafting the manuscript. SSUZ, JTP, HT supported field coordination, MAH, JA assisted supporting the data collection processes. MAH supported with climatic data triangulation. DWB, ERS and JCO contributed to the critical review and refinement of the manuscript. MFH, ERS, UMP and JCO provided strategic oversight and ensured alignment of the study within broader programmatic and policy frameworks. All authors reviewed and approved the final version of the manuscript. CEH is the corresponding author and JCO is the guarantor of the study.

REFERENCES

1. World Health Organization. Scabies [Internet]. Geneva: WHO; [cited 2025 Jul 26]. Available from: https://www.who.int/news-room/fact-sheets/detail/scabies

2. Turan Ç, Metin N, Utlu Z. Epidemiological evaluation of scabies cases encountered in the last three years as a tertiary health center. Turk J Parasitol. 2020 Jun 1;44(2):77–82. doi:10.4274/tpd.galenos.2020.6796

3. Karimkhani C, Colombara DV, Drucker AM, Norton SA, Hay R, Engelman D, et al. The global burden of scabies: a cross-sectional analysis from the Global Burden of Disease Study 2015. Lancet Infect Dis. 2017 Dec;17(12):1247–54. doi:10.1016/S1473-3099(17)30483-8

4. Akpan E, Thean LJ, Baskota R, Mani J, Mow M, Kama M, et al. Costs of primary healthcare presentations and hospital admissions for scabies and related skin infections in Fiji, 2018–2019. PLOS Glob Public Health. 2024 Oct 10;4(10):e0003706. doi:10.1371/journal.pgph.0003706

5. United Nations High Commissioner for Refugees (UNHCR). Country – Bangladesh [Internet]. [cited 2025 Jul 26]. Available from: https://data.unhcr.org/en/country/bgd

6. World Health Organization. WHO implements large-scale ivermectin-based MDA for one million Rohingya refugees [Internet]. 2024 Jan 29 [cited 2025 Jul 26]. Available from: https://www.who.int/bangladesh/news/detail/29-01-2024-who-implements-large-scale-ivermectin-based-mda-for-one-million-rohingya-refugees

7. Usha V, Gopalakrishnannair T. A comparative study of oral ivermectin and topical permethrin cream in the treatment of scabies. J Am Acad Dermatol. 2000 Feb;42(2):236–40. doi:10.1016/S0190-9622(00)90131-2

8. Welch E, Romani L, Whitfeld MJ. Recent advances in understanding and treating scabies. Fac Rev. 2021 Mar 11;10:28. doi:10.12703/r/10-28

9. Mow M, Thean LJ, Parnaby M, Mani J, Rafai E, Sahukhan A, et al. Costs of mass drug administration for scabies in Fiji. PLoS Negl Trop Dis. 2022 Feb 3;16(2):e0010147. doi:10.1371/journal.pntd.0010147

10. Creese A, Parker D. Cost analysis in primary health care: a training manual for programme managers [Internet]. Geneva: WHO; [cited 2025 Jul 26]. Available from: https://files.eric.ed.gov/fulltext/ED380583.pdf

11. UNAIDS. Costing guidelines for HIV prevention strategies [Internet]. [cited 2025 Jul 26]. Available from: https://www.unaids.org/sites/default/files/media_asset/jc412-costguidel_en_2.pdf

12. Xu X, Lazar CM, Ruger JP. Micro-costing in health and medicine: a critical appraisal. Health Econ Rev. 2021 Dec;11(1):1. doi:10.1186/s13561-020-00298-5

13. Médecins Sans Frontières (MSF). Clinical guidelines: diagnosis and treatment manual [Internet]. Geneva: MSF; 2016 [cited 2025 Jul 26]. Available from: https://medicalguidelines.msf.org/en/viewport/CG/english/clinical-guidelines-16686604.html

14. Haacker M, Hallett TB, Atun R. On discount rates for economic evaluations in global health. Health Policy Plan. 2019 Oct 18;czz127. doi:10.1093/heapol/czz127

15. Ashvinbhai PK. A study on evaluating the economical burden of skin diseases: fungal infections and scabies [Internet]. [cited 2025 Jul 26]. Available from: https://doi.org/10.30654/MDJE.10030

16. IOM Bangladesh. IOM Bangladesh appeal 2024: Rohingya humanitarian crisis [Internet]. Cox's Bazar: IOM; 2024 [cited 2025 Jul 26]. Available from: https://bangladesh.iom.int/sites/g/files/tmzbdl1006/files/documents/2024-03/iom-bangladesh-appeal-2024.pdf

17. Joint Response Plan. Rohingya humanitarian crisis 2024 [Internet]. [cited 2025 Jul 26]. Available from: https://rohingyaresponse.org/project/2024-jrp/

18. Hounsome N, Yirgu R, Middleton J, Cassell JA, Fekadu A, Davey G. Cost-effectiveness of mass drug administration for control of scabies in Ethiopia: a decision-analytic model. Front Health Serv. 2024 Sep 18;4:1279762. doi:10.3389/frhs.2024.1279762

19. Chhaiya S, Patel V, Dave J, Mehta D. To study cost effectiveness of topical permethrin versus oral ivermectin in patients of uncomplicated scabies. Int J Basic Clin Pharmacol. 2013;2(6):799. doi:10.5455/2319-2003.ijbcp20131224

20. World Health Organization. Neglected tropical diseases – global [Internet]. [cited 2025 Jul 26]. Available from: https://www.who.int/health-topics/neglected-tropical-diseases

SUPPORTING INFORMATION

S1 File. Detailed cost recording and data analysis tool used for retrospective costing calculations.

 Click here to access/download;Figure;Figure 1. Scenario-wise-cost-comparison.tiff

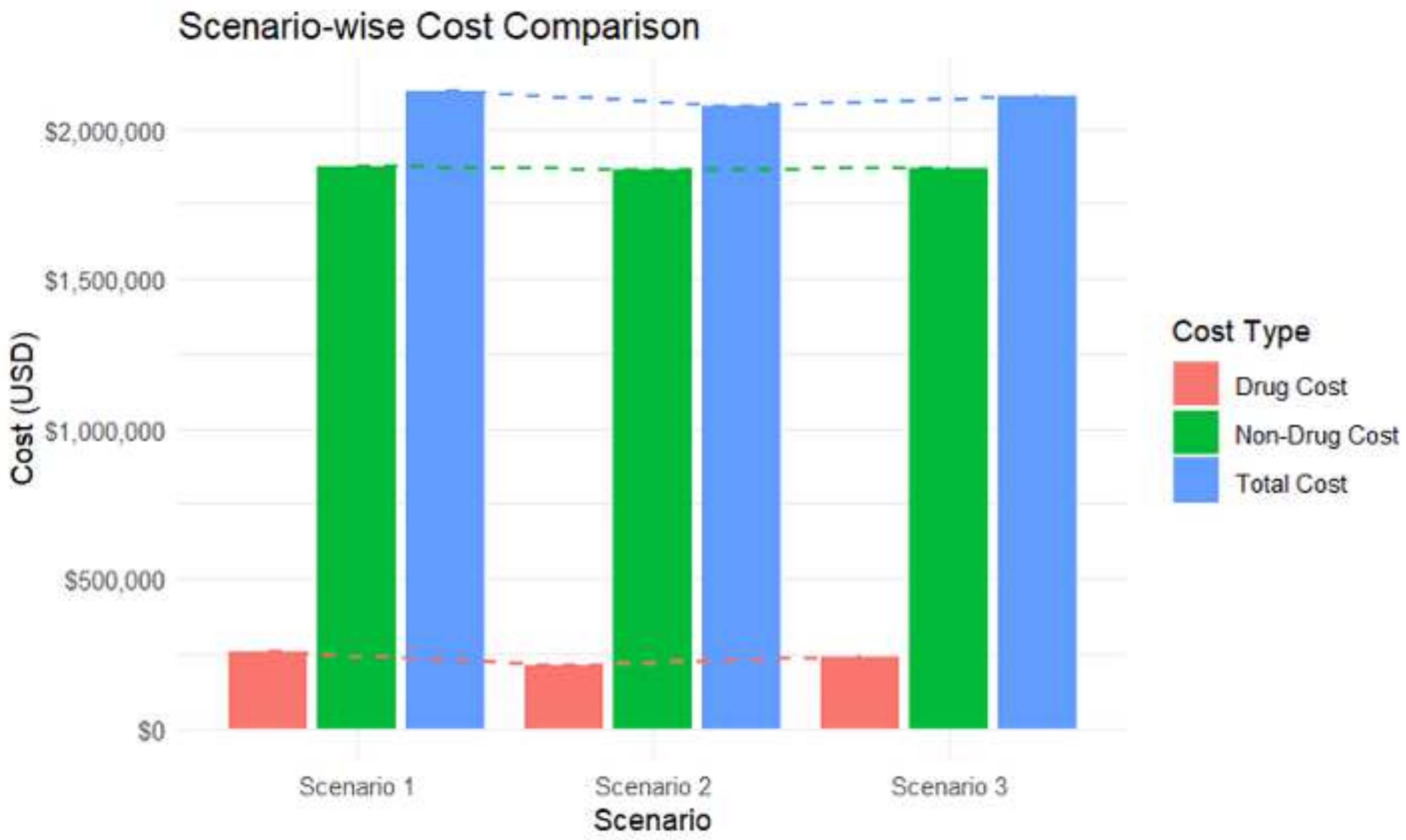

S1 File. Detailed cost recording and data analysis tool used for retrospective costing calculations

Click here to access/download
Supporting Information
S1 File.xlsx

Click here to access/download
Other
017.pdf

Human Subject Research Checklist

Click here to access/download

Other

PLOS_Human_Participants_Research_Checklist_2025.pdf

Economic Impact of a Large-Scale Scabies Upsurge on Healthcare Facilities in Rohingya Refugee Camps: A Retrospective Costing Study

Charls Erik Halder[1]*, Md Abeed Hasan[1], James Charles Okello[1], Sayed Sunny Uz Zaman[1], Julekha Tabassum Poly[1], Hamim Tassdik[1], Dickson Wafula Barasa[1], Emmanuel Roba Soma[1], Md Farhad Hussain[3], U Maung Prue[1], Sirajul Munir Khandokar[1], John Patrick Almedia[2], Jahangir Alam[1]

[1]Migration Health Division, International Organization for Migration (IOM), Bangladesh
[2]Global Migration Health Research & Epidemiology Unit, International Organization for Migration (IOM)
[3]Cox's Bazar Medical College Hospital, Cox's Bazar, Bangladesh

* Corresponding author
Email: cehalder@iom.int

ABSTRACT

Background: Scabies is one of the common infectious skin conditions globally, with a significantly high burden in hot and tropical countries, resource-poor settings, and areas with high population density. Rohingya refugee camps in Cox's Bazar are one of the most protracted refugee crises in the world, sheltering approximately 1,143,096 refugees in 33 overcrowded refugee camps. While most existing literature focuses on mass drug administration (MDA) interventions or community-level estimates, the economic burden of scabies on health system is rarely studied and documented.

Methods: This is a retrospective costing study, where we have used financial and epidemiological data from January 2021 to December 2024. Costing was done from the provider's perspective, focused on what International Organization for Migration (IOM) spent during the period as a health service provider. A combination of standard stepdown approach and micro-costing methods were used. Financial data for the

Formatted: Right: 0.25"

study period 2021 to 2024 were collected from the IOM health programme's annual budget and consumption reports. The study population included all individuals who were clinically diagnosed with scabies and received care at 35 IOM-supported health facilities in Ukhiya and Teknaf, Bangladesh.

Results: The overall estimated financial cost for IOM's scabies outbreak response through its health facilities was USD 2.12 million, with an annual average of USD 531,729. The average cost per scabies management ranged between USD 5.33 and USD 6.54, with the highest in 2021 and the lowest in 2022. Drug costs accounted for 11.92% of the overall cost over 4 years. Of the total cost of $253,629.43 over 4 years, 79% was attributed to permethrin topical cream, which was used to manage an estimated 85% of the total managed cases. Scenario analyses demonstrates that the existing permethrin-based treatment preference is the most expensive treatment modality, compared with ivermectin-based treatment and mixed-treatment approaches.

Conclusion: Although the average cost of treating scabies is relatively low, overall, the treatment cost for such a large population has a significant economic impact. This study found a substantial effect of mass drug administration on reducing the financial burden on the healthcare system.

KEY WORDS

Scabies; Economics; Healthcare Costs; Cost of Illness; Rohingya; Disease Outbreaks; Mass Drug Administration; Communicable Disease Control; Bangladesh

INTRODUCTION

Scabies is one of the common skin conditions globally with significantly high burden in hot and tropical countries, resource poor settings and in areas with high population density. According to World Health Organization, the global incidence of scabies at any given time is more than 200 million, affecting 5 to 50

Formatted: Right: 0.25"

per cent of children in resource-poor areas (1). Refugees and the displaced population possess a higher risk of scabies outbreak due to overcrowded living arrangements, insufficient access to water, sanitation, and laundering facility, limited access to healthcare services and shortage of essential medicines (e.g. scabicides) (2,3).

Scabies is a curable condition, effectively treatable with the topical application of permethrin or oral administration of ivermectin. Unlike non-communicable diseases or high burden communicable diseases, such as, Tuberculosis, HIV and Hepatitis, uncomplicated scabies does not require long-term treatment. The treatment cost of scabies, if considered as single unit, is relatively inexpensive. However, scabies has the high potentiality to cause massive outbreak, especially in overcrowded and refugee settings. Overlooking its collective economic burden in an outbreak setting may create misperception among the policy makers and public health professionals regarding its potential economic impact and the need for adequate planning of resources. For instance, a study in Fiji reported that although the average cost per presentation for scabies at primary healthcare was US$17.7, the estimated annual healthcare costs of scabies and its complications was US$3.0 million equivalent to per capita cost of US$3.3 (4).

The Rohingya refugee camps in Cox's Bazar is one of the largest protracted refugee crises in the world sheltering approximately, 1,143,096 refugees in 33 overcrowded refugee camps (5). Overcrowded living arrangements, fragile shelters, inadequate water, sanitation, and hygiene (WASH) facilities together with heavy monsoons in both the refugee camps and neighboring host communities often result in outbreak of infectious diseases, diphtheria, measles, COVID-19, and Acute Watery Diarrhea (AWD)/cholera. In the first study of our research series on scabies in this setting, we reported a massive outbreak that occurred between 2021 and 2024, with 384,852 cases and an overall attack rate of 5,562.59 cases per 10,000 population over the four-year period (Halder et al.). This high burden increased demand for clinical services and additional resources.~~with a total of 384,852 reported cases with an overall attack rate of 5,562.59 cases per 10,000 population over the four year period (Halder et. al) The high burden of scabies has led to an increased demand for heightened medical attention and additional resources.~~ Simultaneously, it contributed to disruptions in daily life, potentially hindering social interactions and economic activities within the refugee

Formatted: Font: (Default) Times New Roman, 11 pt, Pattern: Clear (White)

Formatted: Font: Not Italic

Formatted: Right: 0.25"

community. In response to the surge of scabies cases, a World Health Organization (WHO)-supported mass drug administration (MDA) campaign was implemented in the Rohingya refugee camps between November 2023 and January 2024. The campaign targeted approximately one million refugees and used oral ivermectin as the primary intervention. The MDA was coordinated with the Government of Bangladesh and implemented through health sector partners operating in the camps, including humanitarian agencies. This large-scale intervention aimed to rapidly reduce transmission and prevent further escalation of the outbreak (6).

Formatted: Font: (Default) Times New Roman, 11 pt, Pattern: Clear (White)

Formatted: Font: (Default) Times New Roman, 11 pt, Pattern: Clear (White)

The disease burden of scabies infestation, along with its associated complications, places a significant financial burden on healthcare systems. It not only impacts the costs associated with medicines (e.g., topical scabicides, antibiotics for secondary infections) and diagnostics but a huge strain over spacing, infection prevention and control, health workforce and quality of service exhausting the available resources of an already overstrained health system (7).

Scabies also can lead to secondary bacterial infection, which could be complicated to life-threatening conditions, like acute glomerulonephritis and rheumatic heart fever, further efforts of healthcare workers and cost of the health system for managing the complications (8). While most existing literature focuses on mass drug administration (MDA) interventions or community-level estimates, economic burden of scabies on health facility is rarely studied and documented (4,9). However, understanding the direct healthcare costs of scabies is critical for effective planning of intervention, resource allocation and initializing cost-effective solutions, like MDA. To address this research gap, this study evaluated the financial impact of the massive upsurge of scabies on healthcare system. This research will assist the health programme and policy makers to understand financial implication of scabies upsurge and thus, facilitate proper planning for effective integration of scabies prevention and management within the essential health service model. This will also assist to examine potential impact and cost effectiveness of new prevention and management strategies or programme.

METHODOLOGY:

Formatted: Right: 0.25"

ETHICS STATEMENT~~AL CONSIDERATION~~

This study was conducted under a pre-approved protocol for secondary analysis of outbreak-related health and programme data. Ethical approval for the study protocol was obtained from the Ethical Review Board of Cox's Bazar Medical College Hospital (CoxMC/2023/017). Administrative permission to use outbreak-response data was obtained from the Office of the Civil Surgeon and the Refugee Relief and Repatriation Commissioner (RRRC), and internal approval was obtained from the International Organization for Migration (IOM) Migration Health Research Unit.

The data analyzed in this study were routinely collected as part of public health outbreak response and health service delivery activities between 2021 and 2024, and were subsequently used for research purposes under the approved secondary-use protocol. Epidemiological case data were extracted from routine outbreak surveillance databases, while financial data were obtained from programme budget and expenditure records. The research team accessed only anonymized, aggregated numerical data and did not have access to identifiable individual-level information at any stage of the study. Given the retrospective nature of the study and the exclusive use of anonymized, aggregated data, individual informed consent was not required.

STUDY SETTING AND POPULATION~~:~~

The study was implemented in Rohingya refugee camps in Ukhiya and Teknaf in Cox's Bazar. The study population included all individuals who were clinically diagnosed with scabies and received care at 35 IOM-supported health facilities in Ukhiya and Teknaf, Bangladesh, between 1st January 2021 and 31st December 2024. Scabies caused a massive upsurge during this period in the refugee camps that prompted a substantial response from IOM. Therefore, these camps were selected for this study to get an in-depth information of the outbreak.

Formatted: Pattern: Clear (White)

Formatted: Space Before: 0 pt, After: 0 pt, Pattern: Clear

Formatted: Font: Not Bold, Not All caps, Pattern: Clear (White)

Formatted: Space Before: 12 pt, After: 8 pt, Pattern: Clear (Background 1)

Formatted: Right: 0.25"

All scabies cases were diagnosed clinically at 35 IOM supported facilities in Ukhiya and Teknaf. MSF clinical guidelines – diagnosis and treatment manual was used for diagnosis and clinical management of the cases. 5% permethrin cream was mainly used for treatment of scabies.

STUDY DESIGN

This is a retrospective costing study, where we have used financial and epidemiological data from January 2021 to December 2024. For this retrospective analysis, financial data and de-identified, aggregated epidemiological data were accessed for research purposes between 05/01/2025 and 20/02/2025. Costing was done from provider perspective focused on what IOM spent during the period as health service provider. A combination of standard stepdown accounting and micro-costing methods were used (10,11).

Micro-costing (or ingredient-based costing) was calculated direct cost per-case by itemizing resources used per patient (12). In this study, we estimated the cost per patient for medicines required, laboratory cost, and supplies for infection prevention and control.

Step-down costing method was used to estimate costing related to capitals, coordination, supervision, operation and maintenance, capacity building, risk communication and community engagement, volunteer payment and salaries and benefits of healthcare workers, meaning that total costs and resources utilized by the whole health programme of IOM during the period was systematically and proportionately distributed to the “specific service unit”. The specific service unit refers, hereby, the scabies consultation and treatment service (13).

The study did not take account of the economic costs (value of resources that could have been productively used elsewhere) such as the value of volunteer time, donated goods, shared infrastructure in the scabies management. All staff and volunteers involved were salaried under the program, and all supplies, equipment, and logistics were directly financed by IOM.

DATA COLLECTION AND MANAGEMENT

Formatted: Right: 0.25"

Financial data for the study period 2021 to 2024 were collected from the IOM health programme's annual budget and consumption reports. A cost recording and data analysis tool was developed to record and analyze all relevant costs associated with scabies response (Supplementary S1 File). Field visits to health facilities and meetings with healthcare workers and medical logisticians were made to develop the cost recording and data analysis tool. All costs were categorized by a) capital investment and b) recurrent costs. Financial and epidemiological data were accessed for research purposes between 05/01/2025 and 20/02/2025 as part of this retrospective analysis. The research team accessed only de-identified, aggregated data and did not have access to any personally identifiable information during or after data extraction and analysis. The research team had access only to deidentified, aggregated data and did not have access to any personally identifiable information during or after data extraction and analysis.

For shared resources, for instance, human resources (healthcare workers and volunteers), coordination, operations and maintenance, and laboratory costs, annual costs were estimated based on the total expenditure reported in the financial documents and relevant procurement orders of the programme. The total costs were then proportionally allocated for scabies using the morbidity burden of scabies in comparison to the total outpatient consultations (Table 1). Scabies response specific investment includes capacity building of healthcare workers and community health workers and development and printing of information, education and communication materials.

Table 1. Year-wise case load and proportional morbidity of scabies

	2021	2022	2023	2024
Scabies	32,046	140,590	150,470	61,746
Infected scabies	2,083	7,348	10,577	2,312
Proportional morbidity (over total consultation)	0.04	0.11	0.12	0.05
**Total outpatient consultations (all causes)**	893,925	1,302,029	1,275,773	1,126,522

Formatted: Right: 0.25"

Note: Proportional morbidity was calculated as scabies consultations divided by total outpatient consultations (all causes) for each year.

Capital costs included biomedical and clinical equipment used for scabies patient management, and costs related to health facility construction/renovation. The costs were annualized using the formula below, assuming the lifespan of construction for 20 years and clinical equipment for 3 years (Creese and Parker, 1994; MSF).

Annual Depreciation Cost = Useful Life (in years)/Purchase Cost of Asset

Coordination costs include salaries of programme staff and office costs at Cox's Bazar and IOM standard 7% overhead cost over the total operation and staff cost. Supervision cost includes salaries of supervisory staff, e.g. clinical supervisors, health facility in-charges and national officers.

The cost of drugs was calculated on per case basis by considering the number of drugs given per patient according to standard clinical protocol and procurement prices as specified in the procurement order.

Data management and cost calculations were performed using Microsoft Excel (Microsoft Corporation, USA). The following assumptions were used to estimate drug and laboratory costs (Table 2). ~~The below assumption was made to determine the drug and laboratory cost.~~ The percentage of case load utilizing a specific medicine was determined by reviewing the consumption report. A 3% discounting rate was used to factor for inflation across year, aligning with WHO guidance (14).

Table 2. Basis and assumptions of calculating costing of drugs and laboratory tests

Category	Type of medicine	% of case load (based on consumption report)	# of items per case	Unit price (BDT)
**Drugs**	Permethrin 5% Ointment	85.00%	2	35
	Ivermectin tab	10.00%	2	10
	Flucloxacillin 500mg cap	5.00%	20	4.8
	Flucloxacillin 250mg cap	2.00%	20	3.1

Formatted: Default Paragraph Font, Font: (Default) Times New Roman, 11 pt

Formatted: Font: (Default) Times New Roman, 11 pt

Formatted: Default Paragraph Font, Font: (Default) Times New Roman, 11 pt

Formatted: Justified

Formatted: Font: (Default) Times New Roman, 11 pt

Formatted: Font: (Default) Times New Roman, 11 pt

Formatted: Right: 0.25"

	Flucloxacillin syp 125mg/5ml	3.00%	1	50
	Cetrizine tab	64.00%	5	0.82
	Cetrizine syp	36.00%	1	9.2
	Benzyl Benzoate 25% ointment	5.00%	0.5	36
**Laboratory tests**	Renal function test, 2% of Infected case, 150 test per unit	0.12%	0.06	2,960
**IPC supplies**	Gloves	100%	2	
	Mask	5%	1	
	Hand Sanitizer (1 bottle per 1000 case)	100%	0.001	

Note: Unit prices are in BDT (Bangladeshi Taka). Permethrin 5% cream is commonly supplied as a 30 g tube; standard instruction was two applications, one week apart, and two tubes were issued per patient visit to support household contact treatment. Flucloxacillin syrup (125 mg/5 mL) is commonly supplied as a 100 mL bottle. Cetirizine tablets are 10 mg per tablet; cetirizine syrup (5 mg/5 mL) is commonly supplied as a 60 mL bottle. IPC unit prices were calculated per item based on procurement unit costs; for reference, common retail unit prices in Bangladesh include gloves (BDT 25 per pair), surgical mask (BDT 5 per piece), and hand rub/sanitizer 500 mL (about BDT 205 per bottle)

Operation and maintenance costs for health facilities included repair and maintenance, rental of land and spaces, generator and utilities, fuel, vehicle maintenance and security cost. Risk Communication and Community Engagement (RCCE) include costing for organizing awareness raising events, focus group

Formatted: Default Paragraph Font, Font: (Default) Times New Roman, 11 pt
Formatted: Font: (Default) Times New Roman, 11 pt
Formatted: Default Paragraph Font, Font: (Default) Times New Roman, 11 pt
Formatted: Font: (Default) Times New Roman, 11 pt
Formatted: Default Paragraph Font, Font: (Default) Times New Roman, 11 pt
Formatted: Font: (Default) Times New Roman, 11 pt
Formatted: Default Paragraph Font, Font: (Default) Times New Roman, 11 pt
Formatted: Font: (Default) Times New Roman, 11 pt
Formatted: Default Paragraph Font, Font: (Default) Times New Roman, 11 pt
Formatted: Font: (Default) Times New Roman, 11 pt
Formatted: Default Paragraph Font, Font: (Default) Times New Roman, 11 pt
Formatted: Font: (Default) Times New Roman, 11 pt
Formatted: Default Paragraph Font, Font: (Default) Times New Roman, 11 pt
Formatted: Font: (Default) Times New Roman, 11 pt
Formatted: Default Paragraph Font, Font: (Default) Times New Roman, 11 pt
Formatted: Font: (Default) Times New Roman, 11 pt
Formatted: Default Paragraph Font, Font: (Default) Times New Roman, 11 pt
Formatted: Font: (Default) Times New Roman, 11 pt
Formatted: Default Paragraph Font, Font: (Default) Times New Roman, 11 pt
Formatted: Font: (Default) Times New Roman, 11 pt
Formatted: Default Paragraph Font, Font: (Default) Times New Roman, 11 pt
Formatted: Font: (Default) Times New Roman, 11 pt
Formatted ...
Formatted: Font: (Default) Times New Roman, 11 pt
Formatted ...
Formatted: Font: (Default) Times New Roman, 11 pt
Formatted ...
Formatted: Font: (Default) Times New Roman, 11 pt
Formatted ...
Formatted: Right: 0.25"

discussions, courtyard sessions, and information, education and communication materials dedicated for scabies prevention and management. Salaries and benefits are counted for healthcare workers engaged in providing outpatient services, including triage, consultation, nursing and medication/pharmacy. Remuneration was costed for community health workers (CHW) engaged in RCCE and facility-based volunteers engaged in triage, counselling and crowd control.

Scenario analyses

A scenario-based costing analysis was conducted to measure the impact of different choice of treatment on the costing of scabies management at health facilities to find out the best approach for cost-efficient treatment of scabies. Scenarios were developed by the public health experts of IOM and based on different proportions of supply availability of permethrin, ivermectin and benzyl benzoate (Table 3). All other capital and recurrent costs were held constant to isolate the effect of different level of supply availability on per-case patient costing.

Table 3. Different planning scenario of cost analyses based on different proportion of supply availability of scabicidal agents

	**Scenario I (Current)**	**Scenario II**	**Scenario III**
	Permethrin-based	Ivermectin-based	Equal permethrin & Ivermectin
**Permethrin**	85%	15%*	47.5%
**Ivermectin**	10%**	80%	47.5%
**Benzyl Benzoate*** **	5%	5%	5%

* Required to manage children <15 kg and pregnant women

** Required to manage complicated cases

*** Kept constant throughout as back up to support in case rupture of both permethrin and ivermectin

Formatted: Right: 0.25"

In current practice (scenario 1), each patient receives two tubes of permethrin at their first visit in the clinic and based on discussion with clinicians, roughly two family members per household visit the clinics. Thus, a family of four members of different age groups roughly receives four permethrin tubes, which are shared across the members for two applications in one week apart. Ivermectin tablets are reserved only for patients with secondary bacterial infections.

In scenarios 2 and 3, since Ivermectin covers regular scabies cases (not only infected), to bring household contacts under treatment one patient is allocated on average 5 Ivermectin tablets of 6 mg with the same assumption that roughly two members per family accesses the health service at health facility.

~~**ETHICAL CONSIDERATION**~~

~~This study was conducted under a pre-approved protocol for secondary analysis of outbreak-related health and programme data. Ethical approval for the study protocol was obtained from the Ethical Review Board of Cox's Bazar Medical College Hospital (CoxMC/2023/017). Administrative permission to use outbreak-response data was obtained from the Office of the Civil Surgeon and the Refugee Relief and Repatriation Commissioner (RRRC), and internal approval was obtained from the International Organization for Migration (IOM) Migration Health Research Unit.~~

~~The data analyzed in this study were routinely collected as part of public health outbreak response and health service delivery activities between 2021 and 2024, and were subsequently used for research purposes under the approved secondary-use protocol. Epidemiological case data were extracted from routine outbreak surveillance databases, while financial data were obtained from programme budget and expenditure records. The research team accessed only anonymized, aggregated numerical data and did not have access to identifiable individual-level information at any stage of the study. Given the retrospective nature of the study and the exclusive use of anonymized, aggregated data, individual informed consent was not required.~~

Formatted: Right: 0.25"

RESULT

Table 4: Estimated retrospective costing analysis for scabies outbreak response (2021 – 2024)

COST CATEGORIES	Type of cost	Costing approach	2021		2022		2023		2024		4 YEARS TOTAL	
			USD	% of total	USD	% of total	USD	% of total	USD	% of total	USD	% of total
**A. CAPITAL COSTS**	Biomedical & Clinical equipment	Step down	$4,587.71	2.19 %	$20,126.88	2.68 %	$21,541.30	2.65 %	$8,839.56	2.49 %	$55,095.46	2.59 %
	Health facility construction/renovation	Step down	$16,687.54	7.96 %	$50,263.58	6.71 %	$54,903.02	6.75 %	$25,514.60	7.19 %	$147,368.75	6.93 %
**SUBTOTAL**			**$21,275.25**	**10.15 %**	**$70,390.47**	**9.39 %**	**$76,444.32**	**9.40 %**	**$34,354.17**	**9.68 %**	**$202,464.21**	**9.52 %**
**B. RECURRENT COSTS**	Supervision	Step down	15,755.52	7.52 %	47,456.30	6.33 %	51,836.61	6.38 %	24,089.58	6.79 %	$139,138.01	6.54 %

Formatted: Font: Bold

Formatted: Right: 0.25"

	Healthcare workers remuneration	Micro-costing	$35,226.57	16.81%	$154,543.56	20.62%	$165,404.15	20.35%	$67,874.29	19.13%	$423,048.56	19.89%
	Volunteer remuneration (CHWs and facility-based volunteers)	Step down	$21,079.00	10.06%	$63,490.84	8.47%	$69,351.18	8.53%	$32,228.97	9.08%	$186,150.00	8.75%
	Drugs	Micro-costing	$21,119.31	10.08%	$92,653.18	12.36%	$99,164.41	12.20%	$40,692.53	11.47%	$253,629.43	11.92%
	IPC supplies	Micro-costing	$8,958.46	4.27%	$39,301.93	5.24%	$42,063.89	5.17%	$17,261.09	4.86%	$107,585.38	5.06%

Formatted: Right: 0.25"

	Laboratory consumables	Micro-costing	$1,922.76	0.92%	$8,435.40	1.13%	$9,028.20	1.11%	$3,704.76	1.04%	$23,091.12	1.09%
	Health facility – operation and maintenance (including repair, rental, utilities, vehicle, fuel)	Step down	$30,973.23	14.78%	$93,292.67	12.45%	$101,903.77	12.54%	$47,356.86	13.35%	$273,526.53	12.86%
	Capacity building	Step down	$2,000.00	0.95%	$4,120.00	0.55%	$4,243.60	0.52%	$4,370.91	1.23%	$14,734.51	0.69%
	Risk Communication and Community Engagement	Step down	$2,916.26	1.39%	$2,938.94	0.39%	$5,832.52	0.72%	$1,035.11	0.29%	$12,722.83	0.60%

Formatted: Right: 0.25"

	(events, sessions, IEC materials)											
**SUBTOTAL**			**$139,951.11**	**66.77%**	**$506,232.82**	**67.53%**	**$548,828.32**	**67.52%**	**$238,614.11**	**67.24%**	**$1,433,626.36**	**67.40%**
SERVICE DELIVERY AND OPERATIONAL EXPENSES (Capital Costs + Recurrent Costs)			$161,226.36	76.92%	$576,623.29	76.92%	$625,272.64	76.92%	$272,968.28	76.92%	$1,636,090.56	76.92%
**C. COORDINATION COST**	Programme staff, office costs and overhead costs (23.08% of service delivery and operational expenses)	Step down	**$48,367.91**	**23.08%**	**$172,986.99**	**23.08%**	**$187,581.79**	**23.08%**	**$81,890.48**	23.08%	$490,827.17	23.08%
**TOTAL ANNUAL COST**			**$209,594.27**	**100.00%**	**$749,610.27**	**100.00%**	**$812,854.43**	**100.00%**	**$354,858.76**	**100.00%**	**$2,126,917.73**	**100.00%**
**COST PER CASE**			**$6.54**		**$5.33**		**$5.40**		**$5.75**		**$5.53**	

Formatted: Right: 0.25"

Table 4 demonstrates the annual cost breakdown of scabies outbreak response from the years 2021 to 2024 at IOM-supported health facilities in the Rohingya refugee camps and host communities in Cox's Bazar. The overall estimated financial cost for IOM's scabies outbreak response through its health facilities was USD 2.12 million with an annual average of USD 531,729.

Aligning with the rise of scabies cases from 2021 to 2023, annual cost of scabies-related health service delivery increased four-fold from USD 209,594 in 2021 to USD 812,854 in 2023. Following the MDA campaign, the cost sharply decreased to $354,858.76 in 2024. The outbreak peak period (2022–2023) had an average annual cost of USD 781,232.35, compared with USD 209,594.27 in the pre-surge year (2021) and USD 354,858.76 in the post-MDA year (2024).

The average cost per scabies management ranged between USD 5.33 to USD 6.54, with the highest in 2021 and lowest in 2022.

Healthcare worker remuneration made up 19.89% of the overall cost, consistently sharing the largest portion of the annual cost across the years, ranging from USD $35,226 (in 2021) to $165,404.15 (in 2023). In addition to this cost, $186,150 was required for the remuneration of community health workers and health-facility-based volunteers, which is another 8 – 10% of the annual cost.

The drug costs consisted 11.92% of the overall cost in 4 years. It increased from USD 19,906.98 (11.37%) in 2021 to a peak of USD 99,164.41 (14.08%) in 2023, before dropping to USD 41,913.31 (14.12%) in 2024. The cost recording and data analysis tool used for the retrospective costing calculations is provided in the Supporting Information (see S1 File)~~The supplementary file 1: details of cost break down provides detail breakdown of the drugs~~. Of the total cost of $253,629.43 in 4 years, 79% of cost was for permethrin topical cream being used for managing an estimated 85% of the total managed cases. On the other hand, tablet ivermectin only accounted for 2.58% of the drug cost, used for management of an estimated 10% of the caseload.

Additionally, an estimated USD 107,585 was utilized for infection prevention and control and USD 23,091 was utilized for laboratory consumables, making up 5% and 1% of the overall cost in 4 years, respectively.

Formatted: Font: 11 pt, English (United States), Ligatures: Standard + Contextual

Formatted: Font: 11 pt, Not Bold, English (United States), Ligatures: Standard + Contextual

Formatted: Font: 11 pt, English (United States), Ligatures: Standard + Contextual

Formatted: Font: 11 pt, Not Bold, English (United States), Ligatures: Standard + Contextual

Formatted: Font: 11 pt, English (United States), Ligatures: Standard + Contextual

Formatted: Font: 11 pt, Not Bold, English (United States), Ligatures: Standard + Contextual

Formatted: Font: 11 pt, English (United States), Ligatures: Standard + Contextual

Formatted: Font color: Auto

Formatted: Font: (Default) Times New Roman, 11 pt

Formatted: Default Paragraph Font, Font: (Default) Times New Roman, 11 pt

Formatted: Font: (Default) Times New Roman, 11 pt

Formatted: Right: 0.25"

Operational and maintenance expenses for health facilities accounted for an estimated 12 to 15% of the annual cost. This proportion was calculated based on the contribution of scabies to the total number of consultations managed by these facilities.

Investment in risk communication and community engagement and capacity building were relatively low throughout the period, each category sharing less than 1% of the overall annual budget.

Table 5. SCENARIO ANALYSIS

	SCENARIO-1	Ratio	SCENARIO-2	Ratio	SCENARIO-3	Ratio
**DRUG COST**	$253,629.43	1	$211,983.90	83.58%	$ 238,718.36	94.12%
**NON-DRUG COST**	$1,873,288.30	1	$1,860,794.64	99.33%	$1,868,814.98	99.76%
**TOTAL COST**	$2,126,917.73	1	$2,072,778.54	97.45%	$2,107,533.34	99.09%

A comparative analysis of different scenarios of proportions of patients treated with topical permethrin and oral Ivermectin.

It demonstrates that existing permethrin-based treatment preference is most expensive treatment modality (Table 51 and Figure 1). Using ivermectin as the preferred treatment (scenario 2) drug costs can be reduced to nearly 83.5% of a permethrin-based approach, saving 2.55% of the overall budget. In case both drugs are utilized in equal proportion (scenario 3), drug costs drop to 94.12% of the current practice with an overall 1% total cost reduction.

Figure 1. Scenario-wise cost comparison of scabies response – Comparison of total, drug, and non-drug costs across three costing scenarios. Drug costs represent expenditures on scabicidal medicines, while non-drug costs include human resources, operations, logistics, coordination, and other programme-related expenditures. All costs are presented in United States dollars (USD).

Formatted: Right: 0.25"

DISCUSSION

This is the first-ever study in the humanitarian and low-resource setting which explored the economic impact of scabies in health system. The study revealed a high economic impact of scabies on the health system valuing USD 2.12 million over four years with an average treatment cost of USD 5.53 per case. The study also found significant impact of mass drug administration on reducing economic burden on health system.

The average cost per scabies management ranged between USD 5.33 to USD 6.54. Since there was no other cost-analysis study available for scabies at humanitarian crises and massive outbreak setting, we could not compare our results with similar settings. However, the cost in our refugee setting was lower than the hospital setting in India and the primary care setting in Fiji, where the estimated average cost per patient were USD 12.20 and USD 17.7, respectively. The cost per patient also seems lower than other health burden, like tuberculosis, HIV, Hepatitis and non-communicable diseases (4,15). However, if we consider the massive scale of the outbreak, the overall financial cost of the scabies upsurge had significant impact on the health system in the low resource setting.

We have found that the estimated financial cost for the IOM's scabies outbreak response was USD 2.12 million from 2021 to 2024 with an annual average of USD 531,729. This annual average is 3.33% of the overall funding appeal made by IOM for in health programme in 2024, indicating a significant economic impact of scabies in health response (16). While IOM covers only one-fourth of the health facilities in the refugee camps, extrapolating it to sector wide response may cost around USD 2.1 million for full coverage of the catchment population in a year, which is around 2.5% of the appeal made by the health sector under the Joint Response Plan in 2025 (17). Our finding coincides with the findings of the country-level recent study of Fiji, where the researchers found although a cost measure of single case of scabies is low, extrapolating the finding across the country, the estimated cost of scabies on the Fijian health system was ~USD 3.0 million per year, equivalent to USD 3.3 per capita (4).

Our study found that annual cost for scabies outbreak response was drastically reduced in 2024 followed by the Mass Drug Vaccination campaign (November 2023 – January 2024), which was 43% of the previous

Formatted: Right: 0.25"

year. Our previous study found that MDA contributed to a significant reduction of scabies cases, which sustained for six months followed by an upward shifting *(Halder et al)*. Aligning with this, our cost-analysis also suggests MDA contributes to significant reduction of economic burden in health systems in high prevalence context. While the estimated cost saving following MDA in IOM facilities was USD 457,996, stimulating it to sector-wide response may lead to a saving of approximately USD 2.0 million with an investment of USD 1M required for the campaign -(6)~~(18)~~. The effectiveness and cost-efficiency of Mass Drug Administration have also been noted in other settings, like Fiji and Ethiopia (9,18). In overcrowded and high burden settings, where the risk factors cannot be addressed properly, by treating the entire community at a time, MDA allows to substantially reduce the scabies prevalence and associated costs. The effectiveness of MDA will only be applicable until the scabies prevalence remains >10% of the community members (18).

We have studied three scenarios to explore the most cost-effective treatment regimen for scabies with first scenario demonstrating the existing practice of permethrin-based treatment, second scenario with ivermectin-based treatment and a third scenario where equal proportion of cases are managed by ivermectin and permethrin. Our analysis found that having ivermectin as preferred treatment (scenario 2) can reduce 16.5% of the drug cost and 2.55% over the overall scabies-related direct and indirect cost. a permethrin-based approach. When both drugs are utilized in equal proportion, 5.88% of drug costs can be reduced, while there will only be a reduction of 1% of the total cost. Topical permethrin and oral ivermectin are both effective and recommended treatment regimen for scabies, although ivermectin has contraindications in case of pregnancy and infants (1,13). A few studies suggest that topical permethrin is clinically more effective than oral ivermectin (7). In India, it was found that although the total cost of treatment with oral ivermectin was lower than topical permethrin, cost for relieving itching and transportation was higher than permethrin (19). In endemic and high-burden setting, ivermectin can be more effective if there remains significant concern on the compliance (18)**.** Therefore, the decision of preferred treatment should not only be decided based on this economic analysis presented here, but also consideration of endemicity, compliance, clinical outcome and response time.

Formatted: Right: 0.25"

This was a retrospective costing analysis, therefore, in case unavailability of exact data, the researchers had to estimate the nearest possible cost based on the discussion on the relevant stakeholders. Another limitation was our study did not measure the economic impact of scabies from the patient and social perspective. For instance, this massive scabies outbreak may have impact on the quality of life and result in social exclusion and stigma and absenteeism at school and workplace (20). There could have costs of scabies related to productivity loss, time of caregiving and travel. Therefore, while further research is warranted to understand the overall impact of scabies on health system and community, prevention and control strategies, including mass drug administration, should be imposed to reduce the burden of scabies on health system and people's lives (1).

CONCLUSION

Scabies has a high impact on health-related quality of life. Our results indicated that, although the average cost of treating scabies is relatively low, overall, the treatment cost for such a large population has a significant economic impact. This study found a substantial effect of mass drug administration on reducing the financial burden on the healthcare system. Strengthening mass drug administration and reinforcing preventive measures is crucial to prevent such an upsurge, especially in low-resourced settings like that of the Rohingya refugee camp.

FUNDING

The authors received no specific funding for this work. The study was conducted as part of routine public health programme activities of the International Organization for Migration (IOM) in Cox's Bazar, Bangladesh. All authors were salaried employees of IOM at the time of the study. No external grants, commercial funding, or institutional research funds were received for this work. The funders had no role in study design, data analysis, decision to publish, or preparation of the manuscript. ~~This study received no specific grant from any funding agency in the public, commercial or not-for-profit sectors.~~

Formatted: Font: (Default) Times New Roman, 11 pt, Ligatures: Standard + Contextual

Formatted: Right: 0.25"

COMPETING INTEREST

The authors declare no competing interests. This study received no external funding. The authors used generative AI tools only for language editing and improving clarity during manuscript preparation. All scientific content, data analysis, and conclusions were developed solely by the authors.

DATA AVAILABILITY

All data underlying the findings of this study and the analysis files are publicly available on Zenodo (DOI: https://doi.org/10.5281/zenodo.17697474). The repository contains fully anonymized, aggregated scabies case counts and the costing inputs used in the economic analysis for 2021–2024. No individual-level or potentially identifiable participant data are shared. Related datasets from the same scabies research series are available on Zenodo (DOIs: https://doi.org/10.5281/zenodo.17648621 and https://doi.org/10.5281/zenodo.17687717).~~All relevant aggregated data and analysis scripts for this article are available from Zenodo at DOI: https://doi.org/10.5281/zenodo.17697474. The dataset includes anonymized, aggregated scabies case counts and climate variables (temperature, rainfall, humidity) for 2021–2023. Related datasets from the same scabies research series are available at DOIs: https://doi.org/10.5281/zenodo.17648621 and https://doi.org/10.5281/zenodo.17687717~~

ACKNOWLEDGMENTS

CEH conceptualized and designed the study, provided overall methodological guidance, and led the drafting and critical revision of the manuscript. MAH assisted in refining the study design and drafting the manuscript. SSUZ, JTP, HT supported field coordination, MAH, JA assisted supporting the data collection processes. MAH supported with climatic data triangulation. DWB, ERS and JCO contributed to the critical review and refinement

Formatted: Font: (Default) Times New Roman, 12 pt, English (United Kingdom)

Formatted: Font: (Default) Times New Roman, 12 pt, English (United Kingdom)

Formatted: Font: (Default) Times New Roman, 12 pt, English (United Kingdom)

Formatted: Font: English (United States)

Formatted: Normal (Web), Right: 0"

Formatted: Font: English (United Kingdom)

Formatted: Normal (Web), Left, Space Before: 0 pt, Line spacing: single

Formatted: Font: 12 pt, English (United Kingdom), Ligatures: None

Formatted: Normal (Web), Left, Right: 0", Line spacing: single

Formatted: Right: 0.25"

of the manuscript. MFH, ERS, UMP and JCO provided strategic oversight and ensured alignment of the study within broader programmatic and policy frameworks. All authors reviewed and approved the final version of the manuscript. CEH is the corresponding author and JCO is the guarantor of the study.

REFERENCES(7)

1. World Health Organization - Scabies [Internet]. [cited 2025 Jul 26]. Available from: https://www.who.int/news-room/fact-sheets/detail/scabies

2. Turan Ç, Metin N, Utlu Z. Epidemiological Evaluation of Scabies Cases Encountered in the Last Three Years as a Tertiary Health Center. Turk J Parasitol. 2020 Jun 1;44(2):77–82. doi:10.4274/tpd.galenos.2020.6796

3. Karimkhani C, Colombara DV, Drucker AM, Norton SA, Hay R, Engelman D, et al. The global burden of scabies: a cross-sectional analysis from the Global Burden of Disease Study 2015. Lancet Infect Dis. 2017 Dec;17(12):1247–54. doi:10.1016/s1473-3099(17)30483-8

4. Akpan E, Thean LJ, Baskota R, Mani J, Mow M, Kama M, et al. Costs of primary healthcare presentations and hospital admissions for scabies and related skin infections in Fiji, 2018–2019. Henao-Martínez AF, editor. PLOS Glob Public Health. 2024 Oct 10;4(10):e0003706. doi:10.1371/journal.pgph.0003706

5. United Nations High Commissioner for Refugees (UNHCR). Country – Bangladesh [Internet]. [cited 2025 Jul 26]. Available from: https://data.unhcr.org/en/country/bgd

6. WHO implements large-scale Ivermectin-based MDA for one million Rohingya refugees [Internet]. 2024 Jan 29. Available from: https://www.who.int/bangladesh/news/detail/29-01-2024-who-implements-large-scale-ivermectin-based-mda-for-one-million-rohingya-refugees

Formatted: Tab stops: 2.45", Left

Formatted: Justified, Line spacing: Double

Formatted: Right: 0.25"

7. Usha V, Gopalakrishnannair T. A comparative study of oral ivermectin and topical permethrin cream in the treatment of scabies☆, ☆☆. J Am Acad Dermatol. 2000 Feb;42(2):236–40. doi:10.1016/S0190-9622(00)90131-2

8. Welch E, Romani L, Whitfeld MJ. Recent advances in understanding and treating scabies. Fac Rev. 2021 Mar 11;10. doi:10.12703/r/10-28

9. Mow M, Thean LJ, Parnaby M, Mani J, Rafai E, Sahukhan A, et al. Costs of mass drug administration for scabies in Fiji. Turner HC, editor. PLoS Negl Trop Dis. 2022 Feb 3;16(2):e0010147. doi:10.1371/journal.pntd.0010147

10. Creese A, Parker D. Cost Analysis in Primary Health Care. A Training Manual for Programme Managers. [Internet]. Available from: https://files.eric.ed.gov/fulltext/ED380583.pdf

11. UNAIDS. Costing Guidelines for HIV Prevention Strategies [Internet]. Available from: https://www.unaids.org/sites/default/files/media_asset/jc412-costguidel_en_2.pdf

12. Xu X, Lazar CM, Ruger JP. Micro-costing in health and medicine: a critical appraisal. Health Econ Rev. 2021 Dec;11(1):1. doi:10.1186/s13561-020-00298-5

13. Médecins Sans Frontières (MSF). Clinical guidelines: Diagnosis and treatment manual. 2016 ed. Geneva: MSF; 2016 [Internet]. 2016. Available from: https://medicalguidelines.msf.org/en/viewport/CG/english/clinical-guidelines-16686604.html#main-content

14. Haacker M, Hallett TB, Atun R. On discount rates for economic evaluations in global health. Health Policy Plan. 2019 Oct 18;czz127. doi:10.1093/heapol/czz127

15. Ashvinbhai PK. A Study on Evaluating the Economical Burden of Skin Diseases: Fungal Infections and Scabies [Internet]. doi:10.30654/MDJE.10030

Formatted: Right: 0.25"

16. IOM Bangladesh. IOM Bangladesh Appeal 2024. Rohingya Humanitarian Crisis [Internet]. Cox's Bazar, Bangladesh; 2024. Available from: https://bangladesh.iom.int/sites/g/files/tmzbdl1006/files/documents/2024-03/iom-bangladesh-appeal-2024.pdf

17. Joint Response Plan - Rohingya Humanitarian Crisis [Internet]. 2024. Available from: https://rohingyaresponse.org/project/2024-jrp/

18. Hounsome N, Yirgu R, Middleton J, Cassell JA, Fekadu A, Davey G. Cost-effectiveness of mass drug administration for control of scabies in Ethiopia: a decision-analytic model. Front Health Serv. 2024 Sep 18;4:1279762. doi:10.3389/frhs.2024.1279762

19. Chhaiya S, Patel V, Dave J, Mehta D. To study cost effectiveness of topical permethrin versus oral ivermectin in patients of uncomplicated scabies. Int J Basic Clin Pharmacol. 2013;2(6):799. doi:10.5455/2319-2003.ijbcp20131224

20. World Health Organization. Neglected tropical diseases – Global [Internet]. [cited 2025 Jul 26]. Available from: https://www.who.int/health-topics/neglected-tropical-diseases

1. World Health Organization. Scabies [Internet]. Geneva: WHO; [cited 2025 Jul 26]. Available from: https://www.who.int/news-room/fact-sheets/detail/scabies

2. Turan Ç, Metin N, Utlu Z. Epidemiological evaluation of scabies cases encountered in the last three years as a tertiary health center. Turk J Parasitol. 2020 Jun 1;44(2):77–82. doi:10.4274/tpd.galenos.2020.6796

3. Karimkhani C, Colombara DV, Drucker AM, Norton SA, Hay R, Engelman D, et al. The global burden of scabies: a cross-sectional analysis from the Global Burden of Disease Study 2015. Lancet Infect Dis. 2017 Dec;17(12):1247–54. doi:10.1016/S1473-3099(17)30483-8

Formatted: Right: 0.25"

4. Akpan E, Thean LJ, Baskota R, Mani J, Mow M, Kama M, et al. Costs of primary healthcare presentations and hospital admissions for scabies and related skin infections in Fiji, 2018–2019. PLOS Glob Public Health. 2024 Oct 10;4(10):e0003706. doi:10.1371/journal.pgph.0003706

5. United Nations High Commissioner for Refugees (UNHCR). Country – Bangladesh [Internet]. [cited 2025 Jul 26]. Available from: https://data.unhcr.org/en/country/bgd

6. World Health Organization. WHO implements large-scale ivermectin-based MDA for one million Rohingya refugees [Internet]. 2024 Jan 29 [cited 2025 Jul 26]. Available from: https://www.who.int/bangladesh/news/detail/29-01-2024-who-implements-large-scale-ivermectin-based-mda-for-one-million-rohingya-refugees

7. Usha V, Gopalakrishnannair T. A comparative study of oral ivermectin and topical permethrin cream in the treatment of scabies. J Am Acad Dermatol. 2000 Feb;42(2):236–40. doi:10.1016/S0190-9622(00)90131-2

8. Welch E, Romani L, Whitfeld MJ. Recent advances in understanding and treating scabies. Fac Rev. 2021 Mar 11;10:28. doi:10.12703/r/10-28

9. Mow M, Thean LJ, Parnaby M, Mani J, Rafai E, Sahukhan A, et al. Costs of mass drug administration for scabies in Fiji. PLoS Negl Trop Dis. 2022 Feb 3;16(2):e0010147. doi:10.1371/journal.pntd.0010147

10. Creese A, Parker D. Cost analysis in primary health care: a training manual for programme managers [Internet]. Geneva: WHO; [cited 2025 Jul 26]. Available from: https://files.eric.ed.gov/fulltext/ED380583.pdf

Formatted: Right: 0.25"

11. UNAIDS. Costing guidelines for HIV prevention strategies [Internet]. [cited 2025 Jul 26]. Available from: https://www.unaids.org/sites/default/files/media_asset/jc412-costguidel_en_2.pdf

12. Xu X, Lazar CM, Ruger JP. Micro-costing in health and medicine: a critical appraisal. Health Econ Rev. 2021 Dec;11(1):1. doi:10.1186/s13561-020-00298-5

13. Médecins Sans Frontières (MSF). Clinical guidelines: diagnosis and treatment manual [Internet]. Geneva: MSF; 2016 [cited 2025 Jul 26]. Available from: https://medicalguidelines.msf.org/en/viewport/CG/english/clinical-guidelines-16686604.html

14. Haacker M, Hallett TB, Atun R. On discount rates for economic evaluations in global health. Health Policy Plan. 2019 Oct 18;czz127. doi:10.1093/heapol/czz127

15. Ashvinbhai PK. A study on evaluating the economical burden of skin diseases: fungal infections and scabies [Internet]. [cited 2025 Jul 26]. Available from: https://doi.org/10.30654/MDJE.10030

16. IOM Bangladesh. IOM Bangladesh appeal 2024: Rohingya humanitarian crisis [Internet]. Cox's Bazar: IOM; 2024 [cited 2025 Jul 26]. Available from: https://bangladesh.iom.int/sites/g/files/tmzbdl1006/files/documents/2024-03/iom-bangladesh-appeal-2024.pdf

17. Joint Response Plan. Rohingya humanitarian crisis 2024 [Internet]. [cited 2025 Jul 26]. Available from: https://rohingyaresponse.org/project/2024-jrp/

Formatted: Right: 0.25"

18. Hounsome N, Yirgu R, Middleton J, Cassell JA, Fekadu A, Davey G. Cost-effectiveness of mass drug administration for control of scabies in Ethiopia: a decision-analytic model. Front Health Serv. 2024 Sep 18;4:1279762. doi:10.3389/frhs.2024.1279762

19. Chhaiya S, Patel V, Dave J, Mehta D. To study cost effectiveness of topical permethrin versus oral ivermectin in patients of uncomplicated scabies. Int J Basic Clin Pharmacol. 2013;2(6):799. doi:10.5455/2319-2003.ijbcp20131224

20. World Health Organization. Neglected tropical diseases – global [Internet]. [cited 2025 Jul 26]. Available from: https://www.who.int/health-topics/neglected-tropical-diseases

SUPPORTING INFORMATION

S1 File. Detailed cost recording and data analysis tool used for retrospective costing calculations.

Formatted: Justified, Line spacing: Double, Numbered + Level: 1 + Numbering Style: 1, 2, 3, … + Start at: 1 + Alignment: Left + Aligned at: 0" + Indent at: 0.25"

Formatted: Font: (Default) +Body (Aptos), 12 pt

Formatted: Normal

Formatted: Normal, Line spacing: Double

Formatted: Font: (Default) Times New Roman, 11 pt, Bold

Formatted: Font: (Default) Times New Roman, 11 pt, Bold

Formatted: Font: (Default) +Body (Aptos), 12 pt

Formatted: Normal, Left

Formatted: Right: 0.25"

Response to Reviewers

Manuscript Title: *Economic Impact of a Large-Scale Scabies Upsurge on Healthcare Facilities in Rohingya Refugee Camps: A Retrospective Costing Study*
Manuscript ID: PGPH-D-25-03807

Dear Editor and Reviewers,

We sincerely thank you for your careful review of our manuscript and for the constructive comments provided. We have carefully addressed each point raised and revised the manuscript accordingly. All changes are reflected in the revised manuscript with track changes enabled.

Below, we provide a detailed, point-by-point response.

Response to Journal Requirement 1: Financial Disclosure Statement

Editor Requirement: Please amend your detailed Financial Disclosure statement to clarify funding sources, grant numbers, role of funders, and salary support.

Response: We have revised the Financial Disclosure statement to comply fully with PLOS Global Public Health requirements. The updated statement now clarifies that no specific grant funding was received for this study and that the work was conducted as part of routine public health programme activities of the International Organization for Migration (IOM), Bangladesh.

We have explicitly stated that:

- No external grants or institutional research funds were received.
- All authors were salaried employees of IOM at the time of the study.
- The funders had no role in study design, data collection and analysis, decision to publish, or preparation of the manuscript.

The revised Financial Disclosure section now reads:

"The authors received no specific funding for this work. The study was conducted as part of routine public health programme activities of the International Organization for Migration (IOM), Bangladesh. All authors were salaried employees of IOM at the time of the study. The funders had no role in study design, data collection and analysis, decision to publish, or preparation of the manuscript."

Response to Journal Requirement 2: Ethics Statement Placement

Editor Requirement: Ensure that the Ethics Statement appears in its entirety at the beginning of the Methods section under the subheading "Ethics Statement."

Response: We confirm that the complete Ethics Statement is now presented at the beginning of the Methods section under the subheading "Ethics Statement." The section includes ethical approval details (CoxMC/2023/017), administrative permissions, clarification of secondary data use, anonymization procedures, and justification for waiver of informed consent.

Response to Journal Requirement 3: Supporting Information Legends

Editor Requirement: Please provide a full list of legends for Supporting Information files after the References section.

Response: We have added a dedicated "Supporting Information" section after the References list, including full legends for all supplementary files:

- S1 File. Detailed cost recording and data analysis tool used for retrospective costing calculations.

Response to Journal Requirement 4: Removal of Identifying Data from Supporting File

Editor Requirement: The file 'Cleaned Dataset_Scabies_v3.xlsx' contained potentially identifying data and was removed. Please anonymize and re-upload an appropriate dataset in compliance with PLOS data policy.

Response: We acknowledge this issue and confirm that the dataset has been removed, as it was not directly relevant to the economic analysis presented in this manuscript. The economic analysis is fully supported by aggregated data and the detailed costing tool provided as S1 File. The Supporting Information files now contain only fully anonymized, aggregated data and do not include any individual-level or potentially identifiable information.

Comment A: Clarify Software Used

Reviewer Comment: Please specify the software used for data management and costing calculations.

Response: We have added the following sentence in the Methods section under *Data collection and management*:

"Data management and cost calculations were performed using Microsoft Excel (Microsoft Corporation, USA)."

Comment B: Duplicate Sentence (Lines 138–139)

Reviewer Comment: Lines 138–139 repeat the same sentence. Please delete one.

Response: We identified the duplicated sentence in the Data Collection and Management section and removed the repetition. The section has been corrected accordingly.

Comment C: Cite Table 2 in Text

Reviewer Comment: Table 2 is not cited in the text. Please add citation after drug costing assumptions.

Response: We have revised the relevant paragraph to include an explicit citation to Table 2. The text now reads:

"The following assumptions were used to estimate drug and laboratory costs (Table 2)."

Comment D: Add Denominator to Table 1

Reviewer Comment: Please include total outpatient consultations to clarify proportional morbidity.

Response: We have added a new row to Table 1:

"Total outpatient consultations (all causes)"

This clarifies how proportional morbidity was calculated. A footnote has also been included:

"Proportional morbidity was calculated as scabies consultations divided by total outpatient consultations (all causes) for each year."

Comment E: Supporting Dataset Clarification

Reviewer Comment: Clarify availability and anonymization of dataset.

Response: We have revised the Data Availability statement to clearly indicate that:

- Only fully anonymized, aggregated data are shared.
- No individual-level or identifiable data are included.
- The dataset and costing inputs are available on Zenodo.
- Related datasets from the same research series are also cited.

The updated Data Availability section now reads:

All data underlying the findings of this study and the analysis files are publicly available on Zenodo (DOI: https://doi.org/10.5281/zenodo.17697474). The repository contains fully anonymized, aggregated scabies case counts and the costing inputs used in the economic analysis for 2021–2024. No individual-level or potentially identifiable participant data are shared. Related datasets from the same scabies research series are available on Zenodo (DOIs: https://doi.org/10.5281/zenodo.17648621 and https://doi.org/10.5281/zenodo.17687717).

Comment F: Data Access Dates

Reviewer Comment:
Please specify dates when data were accessed for research purposes.

Response:
We have added the following sentence in the Study Design section:

"For this retrospective analysis, financial data and de-identified, aggregated epidemiological data were accessed for research purposes between 05/01/2025 and 20/02/2025."

Comment G: Checklist Completion and Formatting

Reviewer Comment: Complete required checklist and formatting updates.

Response:
We have:

- Completed the Human Participants Research Checklist.
- Ensured consistency in table citations.
- Verified internal cross-references.
- Ensured Supporting Information files are correctly labeled.

We sincerely appreciate the reviewers' time and valuable feedback. We believe the revisions have strengthened the clarity, transparency, and methodological rigor of the manuscript. We respectfully submit the revised version for your consideration.

Thank you again for your guidance.

Sincerely,
On behalf of all authors,

Charls Erik Halder
Corresponding Author
International Organization for Migration (IOM), Bangladesh
Email: cehalder@iom.int
