# Supplementary material for: Economic impact of a large-scale scabies upsurge on healthcare facilities in Rohingya refugee camps: A retrospective costing study
Source: PLOS Glob Public Health. 2026 Apr 3;6(4):e0005772. doi: 10.1371/journal.pgph.0005772 (PMC13048477; doi:10.1371/journal.pgph.0005772)
Supplement: Attachment — Submitted filename: Response to Reviewers.docx [file pgph.0005772.s004.docx]

**Response to Reviewers**

Manuscript Title: *Economic Impact of a Large-Scale Scabies Upsurge on Healthcare Facilities in Rohingya Refugee Camps: A Retrospective Costing Study*
Manuscript ID: PGPH-D-25-03807

Dear Editor and Reviewers,

We sincerely thank you for your careful review of our manuscript and for the constructive comments provided. We have carefully addressed each point raised and revised the manuscript accordingly. All changes are reflected in the revised manuscript with track changes enabled.

Below, we provide a detailed, point-by-point response.

**Response to Journal Requirement 1: Financial Disclosure Statement**

**Editor Requirement:** Please amend your detailed Financial Disclosure statement to clarify funding sources, grant numbers, role of funders, and salary support.

**Response:** We have revised the Financial Disclosure statement to comply fully with PLOS Global Public Health requirements. The updated statement now clarifies that no specific grant funding was received for this study and that the work was conducted as part of routine public health programme activities of the International Organization for Migration (IOM), Bangladesh.

We have explicitly stated that:

- No external grants or institutional research funds were received.
- All authors were salaried employees of IOM at the time of the study.
- The funders had no role in study design, data collection and analysis, decision to publish, or preparation of the manuscript.

The revised Financial Disclosure section now reads:

“The authors received no specific funding for this work. The study was conducted as part of routine public health programme activities of the International Organization for Migration (IOM), Bangladesh. All authors were salaried employees of IOM at the time of the study. The funders had no role in study design, data collection and analysis, decision to publish, or preparation of the manuscript.”

**Response to Journal Requirement 2: Ethics Statement Placement**

**Editor Requirement:** Ensure that the Ethics Statement appears in its entirety at the beginning of the Methods section under the subheading “Ethics Statement.”

**Response:** We confirm that the complete Ethics Statement is now presented at the beginning of the Methods section under the subheading “Ethics Statement.” The section includes ethical approval details (CoxMC/2023/017), administrative permissions, clarification of secondary data use, anonymization procedures, and justification for waiver of informed consent.

**Response to Journal Requirement 3: Supporting Information Legends**

**Editor Requirement:** Please provide a full list of legends for Supporting Information files after the References section.

**Response:** We have added a dedicated “Supporting Information” section after the References list, including full legends for all supplementary files:

- S1 File. Detailed cost recording and data analysis tool used for retrospective costing calculations.

**Response to Journal Requirement 4: Removal of Identifying Data from Supporting File**

**Editor Requirement:** The file ‘Cleaned Dataset_Scabies_v3.xlsx’ contained potentially identifying data and was removed. Please anonymize and re-upload an appropriate dataset in compliance with PLOS data policy.

**Response:** We acknowledge this issue and confirm that the dataset has been removed, as it was not directly relevant to the economic analysis presented in this manuscript. The economic analysis is fully supported by aggregated data and the detailed costing tool provided as S1 File. The Supporting Information files now contain only fully anonymized, aggregated data and do not include any individual-level or potentially identifiable information.

**Comment A: Clarify Software Used**

**Reviewer Comment:** Please specify the software used for data management and costing calculations.

**Response:** We have added the following sentence in the Methods section under *Data collection and management*:

“Data management and cost calculations were performed using Microsoft Excel (Microsoft Corporation, USA).”

**Comment B: Duplicate Sentence (Lines 138–139)**

**Reviewer Comment:** Lines 138–139 repeat the same sentence. Please delete one.

**Response:** We identified the duplicated sentence in the Data Collection and Management section and removed the repetition. The section has been corrected accordingly.

**Comment C: Cite Table 2 in Text**

**Reviewer Comment:** Table 2 is not cited in the text. Please add citation after drug costing assumptions.

**Response:** We have revised the relevant paragraph to include an explicit citation to Table 2. The text now reads:

“The following assumptions were used to estimate drug and laboratory costs (Table 2).”

**Comment D: Add Denominator to Table 1**

**Reviewer Comment:** Please include total outpatient consultations to clarify proportional morbidity.

**Response:** We have added a new row to Table 1:

“Total outpatient consultations (all causes)”

This clarifies how proportional morbidity was calculated. A footnote has also been included:

“Proportional morbidity was calculated as scabies consultations divided by total outpatient consultations (all causes) for each year.”

**Comment E: Supporting Dataset Clarification**

**Reviewer Comment:** Clarify availability and anonymization of dataset.

**Response:** We have revised the Data Availability statement to clearly indicate that:

- Only fully anonymized, aggregated data are shared.
- No individual-level or identifiable data are included.
- The dataset and costing inputs are available on Zenodo.
- Related datasets from the same research series are also cited.

The updated Data Availability section now reads:

All data underlying the findings of this study and the analysis files are publicly available on Zenodo (DOI: <https://doi.org/10.5281/zenodo.17697474>). The repository contains fully anonymized, aggregated scabies case counts and the costing inputs used in the economic analysis for 2021–2024. No individual-level or potentially identifiable participant data are shared. Related datasets from the same scabies research series are available on Zenodo (DOIs: <https://doi.org/10.5281/zenodo.17648621> and <https://doi.org/10.5281/zenodo.17687717>).

**Comment F: Data Access Dates**

**Reviewer Comment:**
Please specify dates when data were accessed for research purposes.

**Response:**
We have added the following sentence in the Study Design section:

“For this retrospective analysis, financial data and de-identified, aggregated epidemiological data were accessed for research purposes between 05/01/2025 and 20/02/2025.”

**Comment G: Checklist Completion and Formatting**

**Reviewer Comment:** Complete required checklist and formatting updates.

**Response:**
We have:

- Completed the Human Participants Research Checklist.
- Ensured consistency in table citations.
- Verified internal cross-references.
- Ensured Supporting Information files are correctly labeled.

We sincerely appreciate the reviewers’ time and valuable feedback. We believe the revisions have strengthened the clarity, transparency, and methodological rigor of the manuscript. We respectfully submit the revised version for your consideration.

Thank you again for your guidance.

Sincerely,
On behalf of all authors,

Charls Erik Halder
Corresponding Author
International Organization for Migration (IOM), Bangladesh
Email: cehalder@iom.int
