# Supplementary material for: Economic impact of a large-scale scabies upsurge on healthcare facilities in Rohingya refugee camps: A retrospective costing study
Source: PLOS Glob Public Health. 2026 Apr 3;6(4):e0005772. doi: 10.1371/journal.pgph.0005772 (PMC13048477; doi:10.1371/journal.pgph.0005772)
Supplement: Attachment — Submitted filename: Response to reviewers_R1.docx [file pgph.0005772.s005.docx]

# Response to Editor and Reviewer

**Manuscript ID:** PGPH-D-25-03807R1
**Title:** Economic Impact of a Large-Scale Scabies Upsurge on Healthcare Facilities in Rohingya Refugee Camps: A Retrospective Costing Study

Dear Editor and Reviewer,

We sincerely thank the Academic Editor and Reviewer #1 for the careful evaluation of our revised manuscript. We are encouraged that Reviewer #1 indicated that all previous comments had been adequately addressed and that the manuscript now meets the journal’s publication criteria. In this revision, we carefully addressed the additional editorial comments and revised the manuscript accordingly. All changes have been incorporated in the revised manuscript and are shown in the tracked-changes version.

Below, we provide a point-by-point response.

## **Responses to Journal Requirements**

### Journal Requirement: Reference list review

**Comment:**
Please review your reference list to ensure that it is complete and correct. If you have cited papers that have been retracted, please include the rationale for doing so in the manuscript text, or remove these references and replace them with relevant current references. Any changes to the reference list should be mentioned in the rebuttal letter.

**Response:**
We reviewed the full reference list for completeness, formatting consistency, and accuracy. We confirm that no retracted references are cited in the revised manuscript. We also updated the reference list to improve consistency and added the related scabies outbreak preprint with DOI to replace the earlier non-numbered in-text citation “Halder et al.” with a proper Vancouver-style numbered reference.

## **Responses to Additional Academic Editor Comments**

### Editor Comment 1

**Comment:**
Please see the attached file for additional grammatical and editorial revisions required.

**Response:**
We carefully revised the manuscript throughout for grammar, style, clarity, and consistency in response to the editor’s marked comments. This included correction of sentence structure, wording, capitalization, and formatting inconsistencies across the manuscript.

### Editor Comment 2

**Comment:**
Please see also queries (particularly in the Methods) over treatment types.

**Response:**
We revised the Methods section to clarify the treatment types included in the study. We now specify that scabies cases were managed according to MSF clinical guidelines and local clinical practice; topical permethrin 5% cream was the main treatment for uncomplicated scabies, while oral ivermectin and benzyl benzoate were used in selected scenarios according to clinical indication and availability. We also clarified that flucloxacillin was used where indicated for secondary bacterial infection and cetirizine was used for symptomatic relief of itching.

### Editor Comment 3

**Comment:**
Please clarify the definition of complicated cases.

**Response:**
We added a clear definition in the Methods/Scenario Analysis section. The manuscript now states:

“In this study, complicated cases referred to scabies cases with secondary bacterial infection or other clinical presentations requiring treatment beyond standard topical scabicidal therapy.”

### Editor Comment 4

**Comment:**
Please clarify whether secondary bacterial infections were factored into the cost analysis.

**Response:**
We clarified this explicitly in the Study Design/Methods section. The revised manuscript now states that the costing analysis included both uncomplicated scabies and infected scabies managed at IOM-supported facilities during the study period. We also specify that secondary bacterial infections were factored into the analysis through the infected scabies caseload, related medicine use such as flucloxacillin, and associated laboratory testing assumptions where applicable.

### Editor Comment 5

**Comment:**
Please address wording implying financial burden before the study demonstrates it, and improve the public health framing in the Introduction.

**Response:**
We revised the relevant Introduction paragraph to avoid implying a conclusion before presenting study findings. The revised text now emphasizes the public health and service delivery burden of outbreak response in overcrowded humanitarian settings, including increased demand for medicines, infection prevention and control supplies, staff time, consultation space, and operational resources, rather than prematurely stating a significant financial burden.

### Editor Comment 6

**Comment:**
Please standardize style and terminology, including capitalization and currency format.

**Response:**
We standardized terminology and formatting throughout the manuscript. In particular, we:

- standardized currency reporting to **USD**
- corrected capitalization of terms such as **ivermectin** and **mass drug administration**
- corrected wording such as “Mass Drug Vaccination campaign” to **mass drug administration (MDA) campaign**
- improved consistency across tables and narrative text

### Editor Comment 7

**Comment:**
Please resolve the non-numbered citation “Halder et al.”

**Response:**
We resolved this by citing the related outbreak analysis as a formal preprint with DOI and including it in the reference list in Vancouver style. The previous non-numbered citation has been replaced by a numbered reference in the revised manuscript.

## Response to Reviewer #1

### Reviewer #1

**Comment:**
All comments have been addressed.

**Response:**
We sincerely thank Reviewer #1 for the careful review of the manuscript and for confirming that the previous comments were adequately addressed. We appreciate the constructive feedback provided throughout the review process.

We thank the Academic Editor and Reviewer #1 again for their time and valuable guidance. We believe these revisions have further improved the clarity, transparency, and methodological presentation of the manuscript, and we respectfully resubmit it for consideration.

Sincerely,
**On behalf of all authors**

**Charls Erik Halder**
Corresponding Author
International Organization for Migration (IOM), Bangladesh
Email: [cehalder@iom.int](mailto:cehalder@iom.int)
